# Decreased glucocerebrosidase activity and substrate accumulation of glycosphingolipids in a novel *GBA1* D409V knock-in mouse model

**DOI:** 10.1371/journal.pone.0252325

**Published:** 2021-06-09

**Authors:** Nicole K. Polinski, Terina N. Martinez, Alexander Gorodinsky, Ralph Gareus, Michael Sasner, Mark Herberth, Robert Switzer, Syed O. Ahmad, Mali Cosden, Monika Kandebo, Robert E. Drolet, Peter D. Buckett, Weisong Shan, Yi Chen, Lee J. Pellegrino, Gregory D. Ellsworth, Leo B. Dungan, Warren D. Hirst, Sean W. Clark, Kuldip D. Dave

**Affiliations:** 1 The Michael J. Fox Foundation for Parkinson’s Research, Grand Central Station, New York, New York, United States of America; 2 Taconic Biosciences, Rensselaer, New York, United States of America; 3 The Jackson Laboratory, Bar Harbor, Maine, United States of America; 4 Charles River Laboratories, Ashland, Ohio, United States of America; 5 NeuroScience Associates, Knoxville, Tennessee, United States of America; 6 Saint Louis University, St. Louis, Missouri, United States of America; 7 Merck & Co., Inc., Kenilworth, New Jersey, United States of America; 8 Pfizer, Cambridge, Massachusetts, United States of America; 9 Amicus Therapeutics, Cranbury, New Jersey, United States of America; Azienda Ospedaliero-Universitaria Santa Maria della Misericordia, ITALY

## Abstract

Multiple mutations have been described in the human *GBA1* gene, which encodes the lysosomal enzyme beta-glucocerebrosidase (GCase) that degrades glucosylceramide and is pivotal in glycosphingolipid substrate metabolism. Depletion of GCase, typically by homozygous mutations in *GBA1*, is linked to the lysosomal storage disorder Gaucher’s disease (GD) and distinct or heterozygous mutations in *GBA1* are associated with increased Parkinson’s disease (PD) risk. While numerous genes have been linked to heritable PD, *GBA1* mutations in aggregate are the single greatest risk factor for development of idiopathic PD. The importance of GCase in PD necessitates preclinical models in which to study GCase-related mechanisms and novel therapeutic approaches, as well as to elucidate the molecular mechanisms leading to enhanced PD risk in *GBA1* mutation carriers. The aim of this study was to develop and characterize a novel *GBA1* mouse model and to facilitate wide accessibility of the model with phenotypic data. Herein we describe the results of molecular, biochemical, histological, and behavioral phenotyping analyses in a *GBA1* D409V knock-in (KI) mouse. This mouse model exhibited significantly decreased GCase activity in liver and brain, with substantial increases in glycosphingolipid substrates in the liver. While no changes in the number of dopamine neurons in the substantia nigra were noted, subtle changes in striatal neurotransmitters were observed in *GBA1* D409V KI mice. Alpha-synuclein pathology and inflammation were not observed in the nigrostriatal system of this model. In summary, the *GBA1* D409V KI mouse model provides an ideal model for studies aimed at pharmacodynamic assessments of potential therapies aiming to restore GCase.

## Introduction

Globally, Parkinson’s disease (PD) is the second most common neurodegenerative disease after Alzheimer’s disease and PD is the most common movement disorder. The typical motor symptoms of PD, which present clinically as tremor, rigidity, and bradykinesia, arise from the deficiency of the neurotransmitter dopamine (DA) in the striatum, which in turn occurs as a result of the progressive loss of dopaminergic neuron cell bodies within the substantia nigra pars compacta (SNpc) comprising the main histopathological characterization of PD. Additionally, PD is also characterized by non-motor symptoms (including anosmia, sleep disturbances, gastro-intestinal dysfunction, depression, and cognitive decline) [1] and molecularly by accumulation of alpha-synuclein [2].

The etiology of Parkinson’s disease involves complex gene-environment interactions on the background of aging. Mutations in the gene *GBA1*, which encodes the lysosomal enzyme glucocerebrosidase (GCase), have been linked to PD, and are now recognized to collectively be the greatest known genetic risk factor for development of idiopathic PD [3]. Over 300 mutations in *GBA1* have been identified to date; patients with GCase-associated parkinsonism exhibit varied parkinsonian phenotypes but tend to present with slightly earlier age of onset and higher prevalence of cognitive changes compared to PD patients without *GBA1* mutations [4, 5]. Decreased GCase activity has been reported in PD patients with and without *GBA1* mutations [6]. Accumulating experimental evidence in cell-free systems, cell culture, preclinical animal models, and patient biosamples suggests a correlation between decreased GCase activity and accumulation of the PD-relevant protein alpha-synuclein (aSyn) [7–10]. Thus, GCase has emerged as an important PD-relevant target for understanding the pathogenic mechanisms of parkinsonism and for development of novel therapeutic approaches for disease modifying treatment of PD.

A number of *GBA1* mouse models have been developed and described in the published literature for the lysosomal storage disorder Gaucher’s disease (GD). Rodent models for GD generally feature either knockout of the *GBA1* gene [11–14], chemically-induced models using the inhibitor of GCase conduritol β-epoxide (CBE) [15], or homozygous or compound heterozygous *GBA1* mutations for GD [16, 17]. Similarly, rodent models for investigating the role of the *GBA1* gene or GCase protein in PD have been generated. Similar to the GD lines, these generally feature knockout of the *GBA1* gene [12, 17, 18], intraparynchymal treatment with CBE [19, 20], or heterozygous mutations in the *GBA1* gene [16–18, 21, 22]. However, some of the genetic *GBA1* transgenic mouse models do not exhibit a meaningful accumulation of GCase substrates, particularly in brain.

The *GBA1* D409V point mutation is of particular interest. Although the D409V mutation was described in a study of hetereoallelic patients with GD via cDNA cloning and PCR amplification [23], it has not been identified as causal for human PD or GD [24]. However, this point mutation is of interest as it has been reported to markedly decrease GCase enzymatic activity and concomitantly increase some glycosphingolipid (GSL) substrates. Substantial decreases in GCase activity and GSL increase are observed in peripheral organs of *GBA1* D409V KI mouse models, making this attractive for studying GD [17, 25]. In research focusing on Parkinson’s disease and other synucleinopathies like dementia with Lewy Bodies (DLB), *GBA1* D409V mutant mice exhibit drastically reduce GCase activity, accumulation of the GCase substrate glucosphingosine (GlcSph), accumulation of aSyn in the hippocampus, and learning and memory impairments [17, 18, 21, 26]. These phenotypes are highly relevant for research related to PD, PD dementia, and DLB due to their relevance to human pathology and symptoms [6, 8, 27, 28]. Unfortunately, the previously described *GBA1* D409V transgenic models are not easily accessible to most researchers.

As part of its broader effort to provide the PD research community with crucial preclinical tools and research models, The Michael J. Fox Foundation for Parkinson’s Research (MJFF) sponsors the development, characterization, and distribution of a wide variety of preclinical research tools (www.michaeljfox.org/research-tools-catalog) and has refined a strategy for preclinical animal model generation and characterization [29]. The lack of field-wide access to *GBA1* D409V animal models for PD research was one factor that led to MJFF sponsoring the generation and phenotypic characterization of a new *GBA1* D409V knock-in (KI) mouse model, which is readily available from The Jackson Laboratory repository (www.jax.org/strain/019106), including pharmaceutical and biotechnology companies performing preclinical testing.

Due to its open availability, the *GBA1* D409V KI model at The Jackson Laboratory has been utilized in preclinical studies reported in the published literature. In 2019, two studies reported investigation of PD dementia and DLB-related pathology and phenotypes [30, 31]. Of note, both groups reported a dose-dependent reduction in GCase activity in the hippocampus of homozygous and heterozygous *GBA1* D409V KI mice. Clarke *et al* (2019) went on to characterize cognitive performance and hippocampal pathology in the heterozygous mice at multiple ages, demonstrating cognitive abnormalities, alterations in hippocampal neurochemistry, and neuroinflammation at 12 months of age [30]. Synuclein expression and pathology were not observed in the hippocampus of heterozygous mice up to 12 months of age, but evidence of increased hippocampal aSyn expression in homozygous mice was observed at this time point [30]. Conversely, Burbulla et al (2019) focused on the use of this model in the development of S-181, a small molecule modulator of GCase activity. Within this study, Burbulla and colleagues used the *GBA1* D409V KI mouse model to demonstrate that S-181 can rescue GCase activity deficits, decrease GCase substrate levels, and reduce levels of insoluble aSyn in the hippocampus [31]. Collectively, these studies demonstrate the utility of the *GBA1* D409V model in studying pathology of the hippocampus as it relates to GCase alterations and PD/DLB-related cognitive phenotypes.

Herein, we provide detailed information into the development and phenotypic characterization of a new *GBA1* D409V KI mouse model as it relates to PD-related molecular and motor phenotypes. Thorough characterization of this model with regards to brain and liver GCase expression, activity, substrate accumulation, and lysosomal function are reported. In addition, in depth analysis of nigrostriatal integrity and motor phenotypes are reported to provide investigators with critical information to consider when evaluating the use of this model in therapeutic studies targeting GCase activity or biological studies evaluating the role of GCase deficiency-related phenotypes relevant to PD motor symptoms and pathology.

## Materials and methods

### Generation of the *GBA1* D409V knock-in mouse model

**Fig 1:**
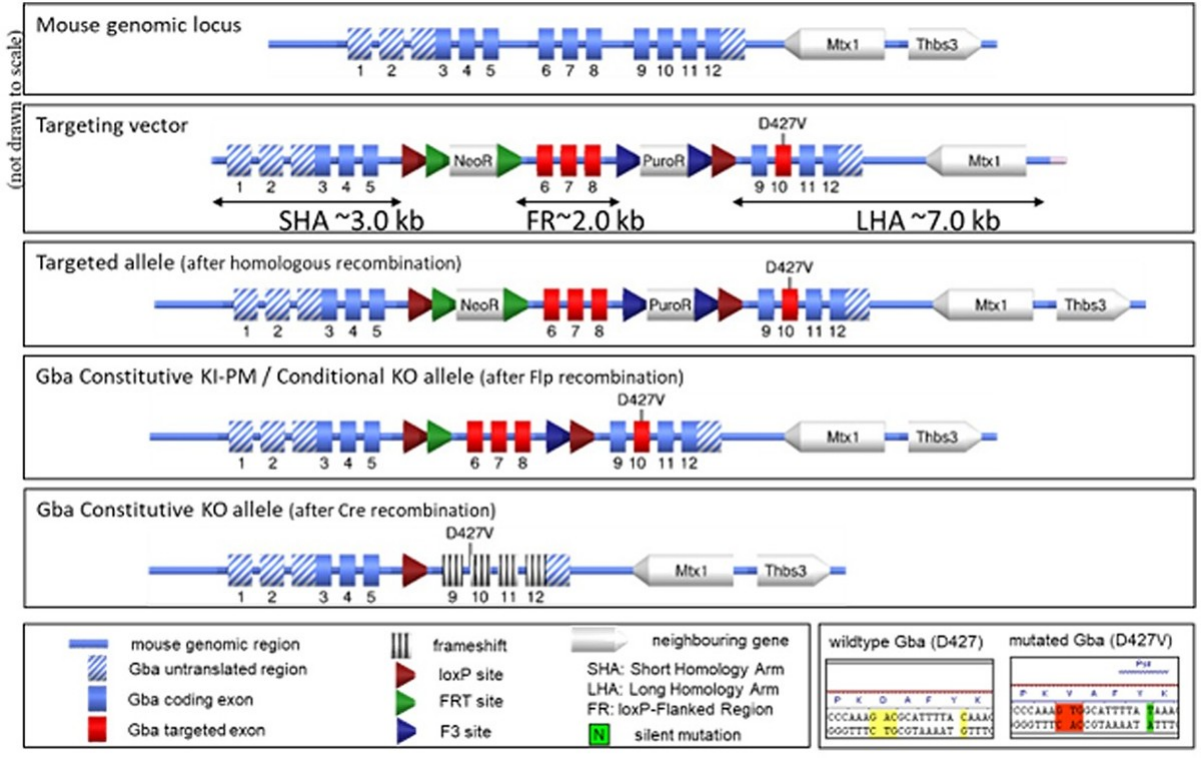
Schematic depiction of *GBA1* targeting strategy. The *GBA1* D409V KI mutation was introduced to the murine *Gba1* gene through the constitutive knockin (KI) of a *Gba1* D427V point mutation as the D427V mutation corresponds to the D409V mutation in the mature GCase protein (Xu et al, 2003). The targeting vector was designed to introduce the D427V point mutation and an additional silent mutation to generate a Psil restriction site for analytical purposes. Positive selection markers were flanked by FRT (Neo^R^) and F3 (Puro^R^) sites respectively. Following homologous recombination in embryonic stem (ES) cells and selection by double positive resistance to neomycin and puromycin, resistant clones were validated for correct integration. Validated ES cells were injected into blastocysts to generate chimers. Chimeric male animals were crossed to FLP recombinase-expressing females to remove the selection cassettes. The resulting animals express the *GBA1* D427V point mutation corresponding to the D409V point mutation in mature protein. An additional feature of this model is the insertion of LoxP sites flanking exons 6–8. After Cre recombination, the murine *GBA1* gene will be constitutively knocked out as a result of loss of gene function through deletion of part of the GCase domain and introduction of a frameshift from exon 5 to all downstream exons with a premature stop codon in exon 9.

The Michael J. Fox Foundation for Parkinson’s Research custom-generated the *GBA1* D409V knockin mouse model in collaboration with Taconic Biosciences for widespread distribution at The Jackson Laboratory as an MJFF Industry Tools Consortium endeavor (www.michaeljfox.org/news/research-tools-consortium). The targeting strategy (**Fig 1**) accommodated constitutive knockin (KI) of a *GBA* D427V point mutation within the locus of the murine *Gba1* gene (NCBI Gene ID 14466; www.ncbi.nlm.nih.gov/gene/14466), located on chromosome 3. The D427V mutation corresponds to the D409V mutation in the mature GCase protein, as described by Xu et al (2003). The targeted point mutation D427V was introduced into exon 10 of the *Gba1* gene using a targeting vector (**Fig 1**) containing a FRT site-flanked Neo^R^ cassette and a F3 FRT site-flanked Puro^R^ cassette. An additional silent mutation was inserted into exon 10 to generate a Psil restriction site for analytical purposes. LoxP sites were inserted to flank exons 6–8 (a region of approximately 2.0 kb) to facilitate Cre-dependent KO of the murine *Gba1* gene by Cre recombinase, resulting in loss of gene function by deleting part of the GCase domain and by generating a frameshift from exon 5 to all downstream exons with a premature Stop codon in exon 9. The targeting vector was generated using BAC clones from a C57BL/6J RPCI-23 BAC library and were electroporated into the TaconicArtemis C57BL/6NTac embryonic stem (ES) cell line. Correctly targeted ES cells were injected into BALB/c blastocysts. The resulting chimeric animals were crossed to FLP recombinase expressing females on the C57BL/6NTac background, C57BL/6NTac-Tg(CAG-Flpe)2Arte, to remove the selection cassettes. This Flp-mediated removal of selection markers resulted in the constitutive KI D409V allele expressing the mutated *Gba1* D427V protein (equivalent to D409V mature protein).

Upon arrival at The Jackson Laboratory, the mice were crossed to C57BL/6NJ (005304, The Jackson Laboratory; www.jax.org/strain/005304) for two generations before a heterozygous x heterozygous breeding colony was established from strain C57BL/6N-Gba^tm1.1Mjff^/J (019106, The Jackson Laboratory; www.jax.org/strain/019106). Genotype was confirmed from a small genomic DNA sample isolated from a tail snip by standard PCR with the mutant gene product = 387 bp, heterozygote = 279 bp and 387 bp, and WT = 279 bp. The forward primer sequence is CAG TTC ACA CAG TGT TGG AGC. The reverse primer sequence is AGG TGA TCG TAT TTC AGT GGC. Additional genotyping information, protocol details (including reaction components and thermocycle parameters) and troubleshooting guidelines can be found by accessing the “Technical Support” tab on The Jackson Laboratory *GBA1* D409V mouse model webpage (www.jax.org/strain/019106).

### Animal use overview

The *GBA1* mouse colony C57BL/6N-Gba^tm1.1Mjff^/J (strain No. 019106) was bred and housed at The Jackson Laboratory mouse repository in a standard barrier mouse pathogen-free vivarium (designated as Area MP23; http://jaxmice.jax.org/health/mp23.pdf) in individually ventilated cages with aspen bedding. The mice had *ad libitum* access to water and food and were fed a standard LabDiet® 5K52 formulation with 6% fat; water was acidified. The mouse colony was maintained under a 12 hr light/12 hr dark cycle. From the heterozygous x heterozygous breeding colony, the mice were pooled at weaning based on date of birth into N = 20 males per cage. Once genotyped prior to 8 wks of age (using the standard PCR genotype procedure described above) the mice were rehoused based on genotype at N = 10/genotype/weaning cage. As the mice aged or signs of hair loss were observed, cage density was dropped to N = 5 per cage for males. If signs of aggression were noted, males were separated and individually housed as needed.

For this study 66 homozygous (hom) *GBA1* D409V KI and 66 wild type (WT) littermate male mice were allocated. The 66 mice per genotype were divided into three equal groups and aged to different timepoints– 4, 8, and 12 months of age (n = 22 mice/genotype/age) with dates of birth ranging no more than plus/minus 3 days for the 4 month cohort and no more than plus/minus 1 week for the 8 and 12 month old cohorts. Within each age group, mice were assigned to one of three groups: Group 1 for biochemistry (n = 7/genotype), Group 2 for neurochemistry (n = 6/genotype), and Group 3 for neurohistology (n = 9/genotype). Assignment to these groups was performed at random for each genotype using a computer program based on stratification of body weights. Group sizes were selected based on previous studies phenotyping models with other PD-related mutations [32].

When each cohort of n = 22 mice/genotype reached the target age, the mice were shipped live to Charles River Labs (formerly WIL Research). At Charles River Labs, viability observations for pain, moribundity, and mortality were performed twice daily and body weight was assessed twice weekly and on the day of scheduled euthanasia. No animals were noted as experiencing pain, significant weight loss, or measures of moribundity during the study. Following a brief period of acclimation (at least one week) mice were examined weekly in a functional observational battery (fully described below) and underwent behavioral testing (see details below) prior to euthanasia and necropsy for phenotyping analyses.

A separate cohort of heterozygous (het) *GBA1* D409V KI mice and WT controls (n = 10/genotype) were obtained from the het x het breeding colony at The Jackson Labs. The het and WT mice were aged to 5 months at which point they were shipped to Amicus Therapeutics for analyses of GCase activity and GSL levels. Finally, an independent cohort of 4 month old WT (n = 8), het (n = 8), and hom (n = 7) mice were obtained from a het x het breeding colony at Merck & Co, Inc (with breeders obtained from the founding Jackson Labs colony) for analysis of *GBA1* mRNA and GCase protein expression.

All animal monitoring and procedures performed at The Jackson Laboratory, WIL Research (now Charles River Laboratory), and Merck & Co met guidelines and regulations of federal, state, and local agencies, as well as the Association for the Assessment and Accreditation of Laboratory Animal Care International (AAALAC). All work was approved by the Institutional Animal Care and Use Committee (IACUC) of that institution. Specifically, the behavioral testing and sacrifice of WT and hom mice for biochemistry, neurochemistry, and neurohistology was approved by the Charles River Ashland IACUC on December 1, 2014 as protocol WIL-784012.

### Necropsy and tissue processing

For sacrifice, animals were perfused in situ with saline and/or sodium cacodylate-based 4% paraformaldehyde at Charles River Laboratory (formerly WIL Research) following deep anesthesia by an intraperitoneal injection of sodium pentobarbital (75 mg/kg).

Group 1 (biochemistry) mice were sacrificed for evaluation of GCase-related functional biology including GCase activity levels, glucosylsphingosine levels, and glucosylceramide levels. Group 1 mice were euthanized and saline-perfused for tissue blood clearance at Charles River Laboratory (formerly WIL Research); brains, livers, and spleens were harvested, weighed and fresh-frozen. For contemporaneous analyses, one brain hemisphere and one liver lobe from each mouse in Group 1 (biochemistry) were shipped (along with spleens) to Amicus Therapeutics and the other brain hemisphere and another liver lobe from the same mice were shipped to Pfizer.

Group 2 (neurochemistry) mice were processed for quantification of dopamine and other neurotransmitters. Group 2 mice were euthanized and saline perfused *in situ*. Brains were rapidly harvested; striatum was macrodissected, weighed, fresh frozen in individual Eppendorf tubes, and reserved at Charles River Labs (formerly WIL Research) for later analyses by UHPLC/MS/MS as described below.

Group 3 (neurohistology) mice were processed for brain histology and stereology. Group 3 mice were euthanized and perfusion-fixed with paraformaldehyde (PFA). Whole brains were harvested, post-fixed, and shipped to NeuroScience Associates for total alpha-synuclein, phospho-serine 129 alpha-synuclein, glial fibrillary associated protein (GFAP), ionized calcium binding adaptor molecule 1 (Iba1), and tyrosine hydroxylase (TH) immunostaining as well as stereology on TH-positive cells in the substantia nigra, as described below.

The 5 month old het and WT mice housed at Amicus Therapeutics for GCase activity and GSL level analyses were euthanized and saline-perfused; tissue was fresh-frozen. The 4 month old WT, het, and hom mice housed at Merck & Co, Inc for *GBA1* mRNA and GCase protein analyses were euthanized and saline-perfused. The frontal cortex was excised for *GBA1* mRNA analysis and the remaining forebrain (immediately behind the frontal cortex that was removed for mRNA) back roughly to the temporal cortex was stored for GCase protein analysis.

### Assessment of *GBA1* mRNA levels by qPCR

mRNA was isolated from frontal cortex tissue using Qiagen RNeasy Mini Prep kit as per manufacturer protocol (Qiagen) and 1 μg of total RNA was reversed transcribed using SuperScript VILO cDNA Synthesis Kit (Invitrogen). Gene expression was determined by quantitative polymerase chain reaction on 7900HT Real Time PCR System (Applied Biosystems) in triplicate wells using Taqman gene expression assays for *GBA1* (Mm0048700_m1 Gba_fam, 4331182; Applied Biosystems) and Glyceraldehyde 3-phosphate dehydrogenase (*GADPH*; Mm99999915_g1, 4352932E; Applied Biosystems). Changes in gene expression were calculated using the comparative C(T) method [33]. Relative *GBA1* mRNA expression was normalized to the values of *GAPDH* mRNA expression and then normalized to the mRNA levels of WT animals. One-Way ANOVA tests were performed for statistical analysis. Individual data points with mean and standard error of the mean are represented on each graph as fold change to WT levels.

### Assessment of total GCase protein expression by immunoblot

The forebrain caudal to the frontal cortex was excised and placed in a 1.5 mL Eppendorf tube containing 250 μL of Radioimmunoprecipitation assay buffer (RIPA; Pierce) supplemented with protease (Roche) and phosphatase inhibitors (Roche) and the samples were homogenized using 5mm stainless steel beads in the TissueLyser at 30 hz frequency for 1.5 minutes (Qiagen). The samples were then centrifuged at 5000 x g for 10 minutes at 4 ^o^C and the supernatant evaluated for Western blot analysis. Protein extracts were denatured in the RIPA buffer containing 1x LDS Sample Buffer (B0007; Invitrogen) and NuPage reducing agent (NP0009; Invitrogen) at 95°C for 5 minutes. After denaturation, 30 μg of protein was loaded on a 4–12% Bis-Tris gel (Invitrogen) and electrophoresed at 150 V for ~90 min. The resolved gel was transferred to PVDF membrane (Invitrogen) using an iBlot standard 7:30 min transfer protocol (Invitrogen). The membranes were blocked for 1 hour with Odyssey Blocking Buffer (LI-COR Biosciences) and then probed overnight at 4 ^o^C with the following primary antibodies in Odyssey Blocking Buffer with 0.1% TWEEN-20: 1:1000 GCase antibody (G4171; Sigma), 1:1000 β-actin (A1978; Sigma). The membranes were washed 3 x 5 minutes with phosphate buffered saline (PBS) containing 0.1% TWEEN-20 at room temperature and then incubated with 1:10,000 IRDye goat anti-rabbit 800 CW (925–32211; LiCor) and 1:1000 IRDye goat anti-mouse 680 CW (925–68070; LiCor) for 1 hour at room temperature. After incubation, the membranes were washed 3 x 5 minutes with PBS containing 0.1% TWEEN-20, followed by 3 x 5 minutes wash with PBS only.

The blots were visualized on LiCor Odyssey (version 3.0.30). Relative fluorescence units for bands associated with GCase and β-actin were calculated by the LiCor Odyssey software for statistical analysis. GCase levels were normalized to β-actin from the same tissue sample and then normalized to the mean levels in WT animals. One-Way ANOVA tests were performed for statistical analysis. Individual data points with mean and standard error of the mean are represented on each graph as fold change to WT levels. Raw images at https://figshare.com/articles/figure/Uncropped_Western_Blots_tif/14611425.

### Assessment of glucocerebrosidase activity

For each of the N = 7 mice per genotype from the Group 1 (biochemistry) aged experimental cohorts described previously, half of the tissue samples (one hemisphere of brain and one lobe of liver) were shipped to Amicus Therapeutics and the other brain hemisphere and liver lobe were shipped to Pfizer, Inc. for independent, contemporaneous analyses. Both brain and liver were divided into 8 samples and frozen at Charles River Labs (formerly WIL Research) prior to analyses. Brain and liver samples of each mouse were prepared in duplicate and assigned a unique sample number such that the 7 mice were treated as 14 blinded samples. Brain hemispheres were thoroughly minced and mixed before sampling. In duplicate, samples were analyzed for GCase activity determination by the conduritol-B epoxide (CBE)/4-Methylumbelliferyl β-D-glucopyranoside (4-MUG) method at Amicus and the MDW941 activity probe method at Pfizer. Moreover, two samples were used for independent lipid extractions for glucosylceramide (GlcCer) analysis, and one sample was used for lipid extraction for GlcSph analysis.

#### CBE/4-MU GCase activity method

These methods apply specifically to the sample preparation and analyses at Amicus for assessment of brain and liver tissue for GCase activity by the CBE/4-MU method. Tissue samples were homogenized in GCase enzyme buffer (McIlvaine citrate/phosphate pH 5.2 containing 0.25% Na-taurocholate and 0.1% TX-100). GCase activity was determined in triplicate from two independent samples of each homogenate, and protein was determined with the BCA assay in singlet from each independent sample of homogenate using BSA as a standard. GCase activity was measured in a 30 min reaction at 37°C in GCase enzyme buffer supplemented to 300 μM N-(n-Butyl) deoxygalactonojirimycin and with or without the covalent GCase inhibitor CBE using 4-MUG as the substrate. CBE-inhibitable GCase activity was converted to nM 4-MU released by comparison with a 4-MU standard curve run with each assay. These levels were normalized to protein weight and hour. Two-Way ANOVA tests were performed for statistical analysis to understand the effect of genotype and age with Bonferroni *post hoc* tests. Individual data points with mean and standard error of the mean are represented on each graph.

#### GCase activity by MDW941 probe method

These methods apply specifically to sample preparation at Pfizer for assessment of brain and liver tissue for GCase activity by the MDW941 probe method. Lysates were prepared by excision of cortex from the frozen brain hemisphere and from tissue obtained from the frozen liver lobe. Tissues were homogenized in 10 volumes of GCase lysis buffer containing 0.25% Triton X-100 using Qiagen Tissuelyser at 25 Hz for 4 min x 2. Samples were then sonicated with a Branson Ultrasonics 450 Digital Sonifier (Branson) for 10 seconds at 80 watts at 20 kHz and diluted to 2 mg/mL with lysis buffer. The GCase activity probe MDW941 was added to the samples to a final concentration of 25 nM probe, 5mM citric acid. Lysate and MDW941 probe were incubated at 37°C for 2 hours. Samples were centrifuged at 21,000 x g for 2 minutes to remove acid precipitates, the supernatant was retained. NuPAGE LDS (4X) sample buffer (ThermoFisher) containing NuPAGE Sample Reducing Agent (dithiothreitol) was added and the mixtures were incubated at 70°C for 10 min. Protein concentration was determined by BCA (Pierce). 30 μg of protein lysate samples were loaded onto a 4–12% gel NuPAGE (ThermoFisher) with TAMRA labelled fluorescent ladder and probe labelled 5 ng recombinant GCase and run at 150 V in MES buffer until the dye front reached the bottom of the gel. A 20% methanol solution was used to wash away unbound probe.

Gels were visualized using a GE Typhoon at 532 nm excitation and 575 nM Long Pass Filter emission, PMT 1000. Bands were quantified for relative fluorescence units by ImageQuant software (GE). Two-Way ANOVA tests were performed for statistical analysis to understand the effect of genotype and age. When significance was identified by the Two-Way ANOVA test, Bonferroni *post hoc* tests were used to understand the significance of the individual comparisons. Individual data points with mean and standard error of the mean are represented on each graph.

### Glycosphingolipid analysis

The following methods were used by Amicus for glycosphingolipid analyses of GlcCer and GlcSph. Liver and brain tissue samples were homogenized in water. Lipid extraction included the addition of the appropriate internal standard for GlcCer and for GlcSph. Briefly, lipid extraction was performed using solid phase extraction. An LC method was employed that separates the more predominant GalCer and GalSph epimers from GlcCer and GlcSph observed in brain tissue. Briefly, isocratic conditions were used with HILIC silica column. Seven GlcCer isoforms were monitored: C16:0, C18:0, C20:0, C22:0, C23:0, C24:0, C24:1. Protein was determined by the BCA assay in triplicate using BSA as a standard.

Levels of GlcCer and GlcSph were normalized to tissue weight. Two-Way ANOVA tests were performed for statistical analysis to understand the effect of genotype and age. When significance was identified by the Two-Way ANOVA test, Bonferroni *post hoc* tests were used to understand the significance of the individual comparisons. Individual data points with mean and standard error of the mean are represented on each graph.

### Immunoblot assessment of Lamp1 levels

Tissue lysates (2mg/mL) from *GBA1* D409V KI hom and WT mouse brain (right hemisphere) and liver (one lobe) from the Group 1 (biochemistry) experimental cohort (N = 7 per genotype per age) described above were processed for Western blot analysis at Pfizer to determine levels of lysosomal-associated membrane protein 1 (Lamp1). Cortex was excised from the brain hemisphere and frozen. Tissue lysates were made in GBA lysis buffer composed of 10 mM Tris, pH 7.5, 250 mM sucrose, 1 mM EDTA, 0.25% Triton X-100. Western blot analysis utilized 4–12% Bis-Tris Midi gels (Thermo Fisher Scientific) with 30 μg protein loaded per well, electrophoresed at 110 V for 100 min. The resolved gel was transferred to nitrocellulose membrane (IB23001; Thermo Fisher Scientific) via dry transfer using iBlot2® Dry Blotting System (Thermo Fisher Scientific). The membranes were blocked for 1 hour with Rockland Blocking Buffer (Rockland) and then probed overnight at 4 ^o^C with the following primary antibodies in Rockland Blocking Buffer with 0.1% TWEEN-20: rabbit monoclonal antibody against Lamp1 (54H11; Cell Signaling) used at 1:2,000 and mouse anti-β-actin monoclonal antibody (A2228; Sigma) used at 1:20,000. The membranes were washed 3 x 5 minutes with Tris buffered saline containing Triton-X100 (TBS-T) buffer and then incubated with 1:10,000 IRDye goat anti-rabbit 800 CW (925–32211; LiCor) and 1:10,000 IRDye goat anti-mouse 680LT (926–68020; LiCor) for 1 hour at room temperature. After incubation, the membranes were washed 3 x 5 minutes with TBS-T buffer.

The blots were visualized on LiCor Odyssey and analyzed using LiCor Odyssey software (version 4.0). Relative fluorescence units for bands associated with Lamp1 and β-actin were calculated by the LiCor Odyssey software for statistical analysis. Lamp1 levels were normalized to β-actin levels from the same tissue sample. Two-Way ANOVA tests were performed to understand the effect of genotype and age. Bonferroni *post hoc* tests were used to understand the significance of the individual comparisons. Individual data points with mean and standard error of the mean are represented on each graph.

### Unbiased stereology

#### Perfusion and tissue processing

Mice in Group 3 (Neurohistology) described above were perfused *in situ* at Charles River Laboratory (formerly WIL Research) following deep anesthesia by an intraperitoneal injection of sodium pentobarbital (75 mg/kg). The mice were first perfused with a sodium cacodylate wash solution (approximately 25 mL) followed by perfusion with a 4% PFA solution in sodium cacodylate (approximately 75 mL). The brains remained in the cranium for approximately 24 hours at 4°C in sodium cacodylate-based 4% PFA. The entire brain (including olfactory bulbs) was then removed, weighed, and the size recorded. The intact brains were placed into sodium cacodylate-based 4% PFA for approximately 24 hours at 4°C, then were transferred into PBS for a minimum of 24 hours and maintained at 4°C until transport. Intact brains were shipped under ambient conditions to NeuroScience Associates using the subject ID without genotype identification to ensure work was performed blinded.

#### Embedding and sectioning

The mouse brains were received at NeuroScience Associates. To prevent freeze artifacts, the brains were treated overnight with 20% glycerol and 2% dimethylsulfoxide prior to being multiply embedded in gelatin matrices using MultiBrain® Technology. After curing, the blocks were rapidly frozen by immersion in isopentane chilled to − 70°C with crushed dry ice, and mounted on the freezing stage of an AO 860 sliding microtome. The MultiBrain® blocks were sectioned in the coronal plane at 40 μm. All sections were collected sequentially into 25 cups per block that were filled with Antigen Preserve solution (49% PBS pH 7.0, 50% ethylene glycol, 1% Polyvinyl Pyrrolidone). Brain sections that were not immediately stained were stored at − 20°C.

#### Immunohistochemistry

For immunohistochemistry, the brain sections were processed and stained free-floating similar to previous published reports [32]. For tyrosine hydroxylase (TH) immunostaining, rabbit anti-TH primary antibody (P40101-0; Pelfreeze) was used at 1:6,000 dilution; secondary antibody was goat anti-rabbit IgG-biotin at 1:238 dilution (BA-1000; Vector Laboratories). For alpha-synuclein staining, mouse anti-aSyn primary antibody (610786; BD Pharmingen) was used at 1:5000 dilution with horse anti-mouse IgG-biotin secondary antibody at 1:238 dilution (BA-2001; Vector Laboratories). For staining of alpha-synuclein phosphorylated at S129, mouse anti-pS129 aSyn primary biotinylated antibody (010–26841; Wako) was used at 1:15,000 dilution. For triple immunofluorescent glial staining, the following primary antibodies were used: rabbit anti-TH primary antibody (P40101-0; Pelfreeze) at 1:1,500 dilution, chicken anti-GFAP primary antibody (CPCA-GFAP, EnCor, Gainesville, FL) at 1:1,500 dilution, and goat anti-Iba-1 primary antibody (ab5076, Abcam) at 1:1,500 dilution. All incubation solutions from the blocking serum onward used TBS-T as the vehicle; all rinses were with TBS.

For chromogen staining, endogenous peroxidase activity was blocked by 0.9% hydrogen peroxide treatment. Following TBS rinses, the sections were immunostained with a primary antibody overnight at room temperature. Vehicle solution contained 0.3% Triton X-100 for permeabilization. Sections were incubated in a biotinylated secondary antibody for two hours at room temperature after rinses. Sections were incubated with an avidin-biotin-HRP complex (Vectastain Elite ABC kit, Vector Laboratories) for one hour at room temperature. Following rinses, the sections were treated with diaminobenzidine tetrahydrochloride (DAB) and 0.0015% hydrogen peroxide to create a visible reaction product, mounted on gelatinized (subbed) glass slides, air-dried, dehydrated in alcohols, cleared in xylene, and coverslipped with Permount. Images were acquired on a TissueScope LE120 from Huron Digital Pathology at 10x resolution (0.8 um/pixel).

For fluorescent staining, sections were immunostained with a primary antibody overnight at room temperature in a vehicle solution contained 0.3% Triton X-100 for permeabilization. Sections were rinsed and incubated in a fluorophore-conjugated secondary antibody for two hours at room temperature. The following secondary antibodies were used: donkey anti-rabbit AlexaFluor 555 secondary antibody (A31572, ThermoScientific) at 1:500 dilution, donkey anti-chicken AlexaFluor 488 secondary antibody (703-545-155, The Jackson Laboratory) at 1:500 dilution, and donkey anti-goat AlexaFluor 647 secondary antibody (A21447, ThermoScientific) at 1:500 dilution. Sections were washed twice in 1 min increments in 50% ethanol followed by several washes in TBS. Sections were mounted on SuperFrost Plus slides and coverslipped with Vectashield mounting medium (Vector Laboratories). Images were acquired on an Olympus VS200 WSI system (Olympus Life Science, Waltham, MA) at 20x resolution (0.325 μm/pixel).

#### Stereological estimates of cell number

The unbiased stereology method and validation is previously published and described in detail [34, 35]. Briefly, a Nikon Eclipse E800 microscope, connected with a IMI Tech Color Digital Video Camera, which operated an Advanced Scientific Instrumentation MS-2000 motorized stage input into a Dell Precision 650 Server and a high resolution plasma monitor was used in tandem with the *Stereologer* software package (Stereology Resource Center, Baltimore INC) to estimate neuronal number. Using design-based stereology, the number of TH-positive neurons was quantified in the SNpc of the mice. For the estimation of neuronal number, every 8^th^ section containing the SNpc (− 4.56 to − 6.60 mm from Bregma) was selected from a random initial sort, described as systemic-random sampling. The section sampling fraction (ssf) was 1/6 [36]. In design-based stereology, the optical disector method affords an equal probability for each neuron to be counted within the estimated total number of neurons. The counting process was as follows: first, each mouse brain coronal section was visualized at low magnification (4 x) and the region of interest (ROI) was precisely outlined in reference to a stereotaxic atlas of the mouse brain. Systematic random grids to select counting frames within the ROI were then applied by the software. In each counting frame, TH-positive neurons were counted at high magnification with a 100 ×/1.4 aperture oil immersion lens (yielding 3600 ×) by the optical disector principle, utilizing the disector probe [37] in combination with optical sectioning of the z-axis (or optical fractionator method) [36]. According to the counting rule of the optical disector, a neuron was counted only if its nucleoli could be resolved in focus within the counting frame boundaries without touching the exclusion lines. The top and bottom of the sample tissue–designated as a guard volume–were excluded, avoiding a blade sectioning artifact effect termed “capping”, or cutting the neurons in half. In this present study, the guard zone, as measured by a linear encoder, was a minimum of 3 μm. The middle 10–12 μm area of an 18–21 μm thick sample was analyzed for counting. Section thickness was individually determined by a linear encoder and the mean thickness of count frames measured by a 100 ×/1.4 aperture oil immersion lens. After every sample coronal brain section was analyzed, the total number of TH-positive neurons in the interest area (N) was estimated by multiplying the number of counted neurons (ΣQ) by the reciprocals of three sample fraction, such as the section sampling fraction (ssf), the area sampling fraction (asf), and the section thickness sampling fraction (stsf). The *Stereologer* software package (Stereology Resource Center, Baltimore INC, Baltimore, MD) calculated the total neuronal number according to the following equation:

N=ΣQx(1/ssf)x(1/asf)x(1/stsf)

The average number of slides per animal varied from 5–7 and the Coefficient of Error (CE) was capped at 0.15 with the actual mean value of 0.96.

Two-Way ANOVA tests were performed for statistical analysis to understand the effect of genotype and age. Individual data points with mean and standard error of the mean are represented on each graph.

### Neurochemistry to measure striatal neurotransmitters

For each of the N = 6 mice per genotype from the Group 2 (neurochemistry) aged experimental cohort described previously, dopamine, its metabolites, and other relevant neurotransmitters were measured by the Charles River Bioanalytical Chemistry Department (formerly WIL Research) in a blinded fashion according to the Laboratory Method for the Analysis of Dopamine (DA), Serotonin (5-HT), 3,4-dihydroxyphenylacetic acid (DOPAC), Homovanillic acid (HVA), 5-hydroxyindole-3-acetic acid (5-HIAA), and Norepinephrine (NE) in Mouse Brain Homogenate by LC/MS/MS (Lab Method No: 784005A.MT).

#### Striatal tissue processing and extraction

The left hemisphere of the grossly dissected and weighed striatal tissue was analyzed for neurochemistry; right hemispheres were reserved for future analysis or follow up studies. Frozen samples in Eppendorf tubes were placed on wet ice but were not allowed to fully thaw. Chilled 0.1% formic acid in Milli-Q water (FA-MQ) was added to each tissue sample in a ratio of 9:1 (v:v, volume [FA in MQ]:mass brain tissue). Three 4-mm acid washed silica beads (BAWG 4000-2000-18; OPS Diagnostics) were quickly added to each sample tube, which was recapped and placed in a 15 mL tube and stored on wet ice. Samples were then homogenized in the Geno/Grinder® 2010 (SPEX SamplePrep) automated tissue homogenizer for 1 min at 1,3000 rpm followed by centrifugation at 4,000 rcf at 4°C. Samples were extracted immediately following homogenization and remaining homogenate was stored at -70°C.

For sample extraction, a 96-well plate on wet ice was used. A volume of 0.025 mL of FA in MQ was added to each well designated to contain a matrix blank sample; 0.025 mL of Internal Standard Working Solution (ISWS, 100 ng/mL each of DA-d4, 5-HT-d4, NE-d6 and 200 ng/mL each of DOPAC-d5, HVA-d5, 5-HIAA-d5 brought to volume with chilled FA-MQ) was added to each well designated to contain a calibration, quality control (QC), blank with internal standard (IS), or experimental sample. A volume of 0.175 mL of chilled FA in MQ was then added to each sample well. 0.025 mL of each calibration, QC, blank, and experimental sample was transferred into its respective well of the 96 well extraction plate. The plate was covered and vortex-mixed for 2 min at 1,500 rpm followed by centrifugation at 3,500 rpm for 5 min. To complete the dilution-filtration sample extraction, a volume of 0.200 mL of the supernatant was then loaded into a Whatman UNIFILTER 96-well Microplate—2 mL SPE plate (7720–7236; Whatman) using a multichannel pipette (Rainin) and was pushed through the filter to a clean collection plate. The collection plate was then centrifuged at 3,500 rpm for 5 min at 4°C. Processed samples were stored at 4°C in the sample compartment of the LC instrument or in the refrigerator until analysis.

#### System suitability testing and calibration standards

A system suitability test (SST) was used, consisting of a minimum injection of sample solution three consecutive times meeting the following criteria: ≤ 5% variability in retention time of each system suitability injection as compared to the mean retention time of the system suitability injections; mean analyte peak signal:noise ratio ≥ 3; and a relative standard deviation ≤ 10% in the response of the analyte peak area count or peak area ratio. Standard calibration samples were fresh-prepared on wet ice on the day of use. Standards calibration samples were diluted in FA-MQ to a final volume of 5.0 or 1.0 mL and ng/mL concentrations of 2,400.00, 1,200.00, 1,080.00, 600.00, 300.00, 120.00, 60.00, 12.00, for DA and 5-HT; final ng/mL concentrations of 5-HIAA, NE, DOPAC and HVA were 2x higher. A final sample injection volume of 5–10 μL was used for UHPLC/MS/MS analysis of experimental samples.

#### UHPLC/MS/MS analyses

Instrumentation/detection was UHPLC/MS/MS (API 4000, ESI+) for DA, NE, 5-HT, and 5-HIAA and was UHPLC/MS/MS (API 4000 ESI-) for DOPAC and HVA. Standard curve ranges were 6.00–1200 ng/mL for DA and 5-HT and 12.0–2400 ng/mL for DOPAC, HVA, 5-HIAA, and NE. For UHPLC positive ion mode analysis, a Waters Acquity® UPLC instrument equipped with autosampler and a Restek PFP Propyl, 50 x 2.1 mm, 1.9-μm particle size column (9419252; Restek) at 60°C was used. Positive mode sample injection and analysis was done before negative mode analysis due to compound instability and was done the day of sample extraction and preparation. Run time was 4 min, and the autosampler temperature was 4°C. Mobile phase A (MPA) was FA-MQ, which was also used as weak needle wash solution. Mobile phase B (MPB) was 0.1% acetonitrile with formic acid (ACN-FA, 1000:1 v/v), which was also used as strong needle wash solution. Seal wash solution was 90:10 (v/v) Milli-Q water and acetonitrile (MQ-ACN). The gradient program was time in minutes: initial = 0, 0.50, 2.60, 2.70, 3.30, 3.31, 4.00 with corresponding flow rates (mL/min) of 0.400, 0.400, 0.400, 1.000, 1.000, 1.000, 1.000, and corresponding MPA % of 100, 100, 70, 10, 10, 100, 100 and MPB % of 0, 0, 30, 90, 90, 0, 0.

Mass spectrometer parameters for positive ion mode utilized an Applied Biosystems/MDS Sciex API 4000™ triple quadrupole instrument with a Turbo Spray, positive-ion mode interface and a multiple reaction monitoring scan mode. Curtain gas was 20.0, gas setting 1 was 60.0, gas setting 2 was also 60.0, ionization voltage was 5500 V, temperature was 550°C, collision gas setting was 8.00, entrance potential was 10.90, and interface heater was on.

For UHPLC negative ion mode analysis, a Waters Acquity® instrument with Waters UPLC HSS T3, 50 x 2.1 mm, 1.8 μm particle size column (176001131 or 186003538; Waters) at 60°C was used. Run time was 4 min, and the autosampler temperature was 4°C. MPA was 0.1% glacial acetic acid in Milli-Q water (GAA-MQ), which was also used as weak needle wash solution. MPB, also used as strong needle wash solution, was 0.1% GAA in ACN (GAA-ACN). Seal wash solution was the same for positive and negative ion mode analyses (90:10 (v/v) MQ-ACN). The gradient program for negative ion mode was time in minutes: initial = 0, 2.50, 2.60, 3.20, 3.21, 3.30, 4.00 with corresponding flow rates (mL/min) of 0.400, 0.400, 0.400, 1.000, 1.000, 1.000, 1.000, and corresponding MPA % of 100, 75, 5, 5, 5, 100, 100 and MPB % of 0, 25, 95, 95, 95, 0, 0.

Mass spectrometer parameters for negative ion mode utilized an Applied Biosystems/MDS Sciex API 4000™ instrument with a Turbo Spray, negative-ion mode interface and a multiple reaction monitoring scan mode. Curtain gas was 20.0, gas setting 1 was 60.0, gas setting 2 was also 60.0, ionization voltage was -4200 V, temperature was 550°C, collision gas setting was 8.00, entrance potential was -10.0, and interface heater was on.

Two-Way ANOVA tests were performed for statistical analysis to understand the effect of genotype and age. When significance was identified by the Two-Way ANOVA test, Bonferroni *post hoc* tests were used to understand the significance of the individual comparisons. Individual data points with mean and standard error of the mean are represented on each graph.

### Animal behavior

Behavioral analyses were performed on all mice housed at WIL Research (now Charles River Laboratories). Each aged experimental cohort of mice (at 4, 8, and 12 mo of age) were assessed for behavior prior to sacrifice and additional molecular/biochemical/histological analyses.

#### Functional observational battery

To evaluate general behavioral phenotypes in the mice, the noninvasive Functional Observation Battery (FOB), a collection of assessments used to detect gross functional deficits, was performed weekly on all mice. The FOB consists of a series of tests categorized into the following domain observations: home cage (including but not limited to: posture, convulsions, tremors, and eyelid closure), handling (including but not limited to: ease of removal from cage, lacrimation, salivation, piloerection, respiratory rate, mucous secretions, and muscle tone) open field (including but not limited to: time to first step, rearing, mobility, grooming, gait/gait score, convulsions, tremors, arousal, bizarre/stereotypic behavior) sensory (inclusive of: approach, touch, and startle responses, tail pinch, olfactory orientation, pupil and eyeblink response, forelimb/hindlimb extension, and air righting reflex) neuromuscular (inclusive of: hindlimb extensor strength, grip strength–hindlimb and forelimb, hindlimb foots splay, rotarod performance, and pole climb test) and physiological (catalepsy, body temperature, and body weight) [38]. Neurobehavioral endpoints were conducted blinded and animals underwent further randomization into study replicates using a computer program, with each group equally represented in each replicate. Two-Way ANOVA tests were performed for statistical analysis.

### Statistical analysis

In all cases, the experimental unit is a single animal and data is represented as mean ± standard error of the mean (SEM). Statistical analyses and graphing were performed using the GraphPad Prism Software (version 7.05). Outliers were identified using the GraphPad Prism Software using the ROUT method. Only one outlier was identified and removed (a 4 month old hom *GBA1* D409V KI mouse in the brain GlcCer analysis). As the outlier appeared to be due to a technical issue, the animal was not excluded from other analyses. Distribution normality was tested using the Shapiro-Wilk test. In cases where datasets had non-normal distributions, Mann-Whitney tests were used in place of T-tests. Raw data is available within S1 File.

## Results

### The *GBA1* D409V KI model is viable and vital

The *GBA1* D409V KI targeting strategy (**Fig 1**) resulted in a viable mouse model constitutively expressing the human *GBA1* point mutation D409V knocked into the mouse *Gba1* locus. No gross differences between the *GBA1* D409V KI and WT mice of the same background strain were observed. Normal breeding behavior, litter sizes, weaning behavior, development, and onset of sexual maturity were evident in het and hom *GBA1* D409V KI mice. The *GBA1* D409V KI founders were bred into distinct experimental cohorts and evaluated at different ages for model characterization and phenotyping. While we focused our phenotyping studies predominately on mice homozygous for the *GBA1* D409V KI mutation, we also investigated effects in mice heterozygous for the same mutation to interrogate potential gene dosing effects and because PD patients with *GBA1* mutations are typically heterozygous. Importantly, the aged *GBA1* D409V KI and WT mouse cohorts were obtained at different times and were not analyzed longitudinally. Within a given aged cohort however (i.e. 4, 8, or 12 months of age) the *GBA1* D409V KI and WT mice were contemporaneously bred, housed, and experimentally evaluated.

### Baseline *GBA1* mRNA expression levels in *GBA1* D409V KI mice are unchanged

**Fig 2:**
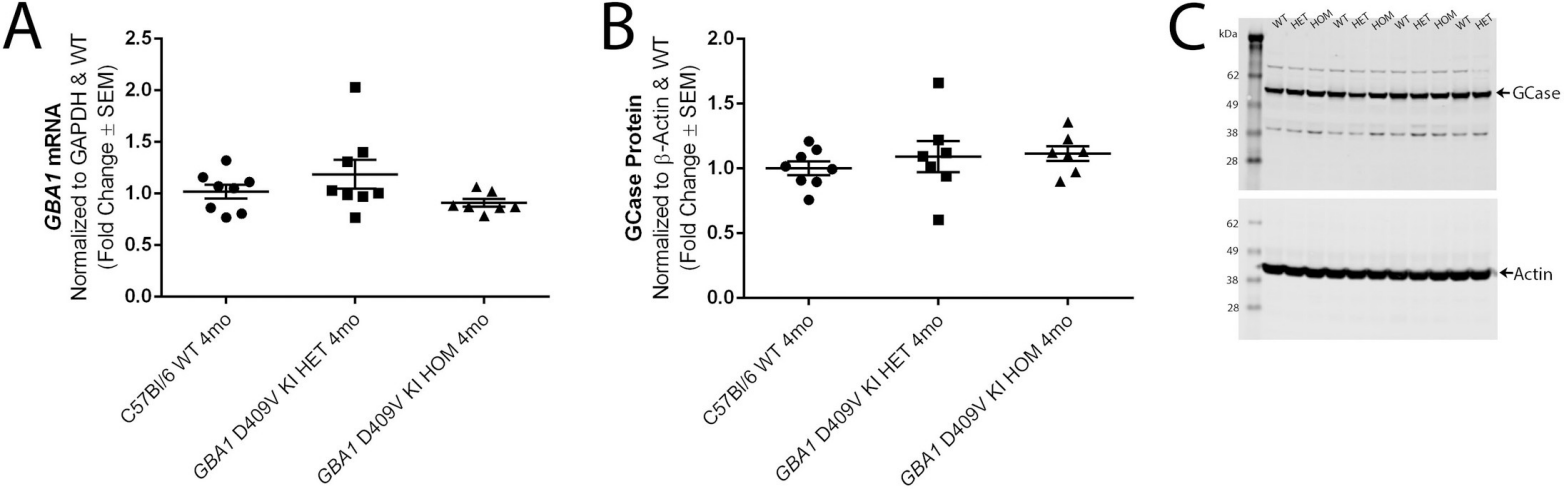
Introduction of the D409V point mutation to the *GBA1* gene does not affect *GBA1* mRNA transcription or GCase protein translation. (A) *GBA1* mRNA levels assessed by qPCR in frontal cortex tissue from 4 month old C57Bl/6 wild type (WT) mice, *GBA1* D409V KI heterozygous (HET) mice, and *GBA1* D409V KI homozygous (HOM) mice. *GBA1* mRNA levels are normalized to *GAPDH* as the housekeeping gene, with graphs depicting fold changes relative to WT expression levels. *GBA1* mRNA levels are consistent between groups with no significant differences observed (n = 8/group; *p* > 0.05). WT levels measure 1.016±0.068, HET levels measure 1.185±0.140, HOM levels measure 0.9066±0.037. (B-C) GCase protein levels assessed by Western blot in forebrain tissue (caudal to frontal cortex) from the same mice used for mRNA analysis. (B) GCase protein levels are normalized to β-actin as the housekeeping protein, with graphs depicting fold changes relative to WT GCase protein expression levels. GCase protein is unchanged in the HET and HOM *GBA1* D409V KI mice as compared to WT (n = 7-8/group; *p* > 0.05). WT levels measure 1.000±0.052, HET levels measure 1.091±0.120, HOM levels measure 1.114±0.057. (C) Representative image of Western blot results for alternating samples of WT, HET, and HOM *GBA1* D409V KI mice. Abbreviations: SEM, standard error of the mean; WT, wild type; *GBA1*, gene encoding human glucocerebrosidase; GCase, glucocerebrosidase; KI, knockin; HET, heterozygous; HOM, homozygous; mo, month.

We first wanted to assess the effect of the *GBA1* KI mutation on *GBA1* mRNA. For this analysis, frontal cortex tissue was selected given the high levels of expression of *GBA1*, the importance of this region in patients and models with *GBA1* mutations [12, 27], and reports of the interplay of GCase and aSyn in this region [21, 28]. The comparative C(T) method of qPCR evaluation was used to assess *GBA1* mRNA levels at baseline in brain tissue from WT, het, and hom mice at 4 months of age. Interestingly, no significant difference in *GBA1* mRNA expression was observed in mice heterozygous and homozygous for the D409V KI mutation compared to *GBA1* mRNA levels WT mice at the same age (F(2,20) = 2.115, *p* = 0.1468; **Fig 2A**).

### Baseline GCase protein expression levels in *GBA1* D409V KI mice are unchanged

To evaluate the effects of knocked in *GBA1* D409V mutation on baseline expression of GCase protein, we assessed total GCase protein levels by immunoblot in the same mice that were analyzed for *GBA1* mRNA levels (**Fig 2A**). Forebrain tissue homogenate from 4 month old cohorts of het and hom *GBA1* D409V KI and WT mice had equivalent GCase protein levels across all genotypes (F(2,19) = 0.5870, *p* = 0.5657; **Fig 2B and 2C**) similar to results for *GBA1* mRNA (**Fig 2A**).

### GCase enzyme activity is decreased in brain and liver from *GBA1* D409V KI mice

The functional role of GCase is lysosomal hydrolysis of the glucosphingolipids GlcCer and GlcSph. In addition to the observed decrease in GCase activity in PD patients with mutations in *GBA1* [6], several publications have independently reported decreased GCase activity in animal models harboring *GBA1* mutations [10, 17, 18, 21, 39], including the *GBA1* D409V mutation. All together, these data demonstrate the importance of evaluating GCase enzymatic function beyond baseline *GBA1* mRNA and GCase protein levels.

**Fig 3:**
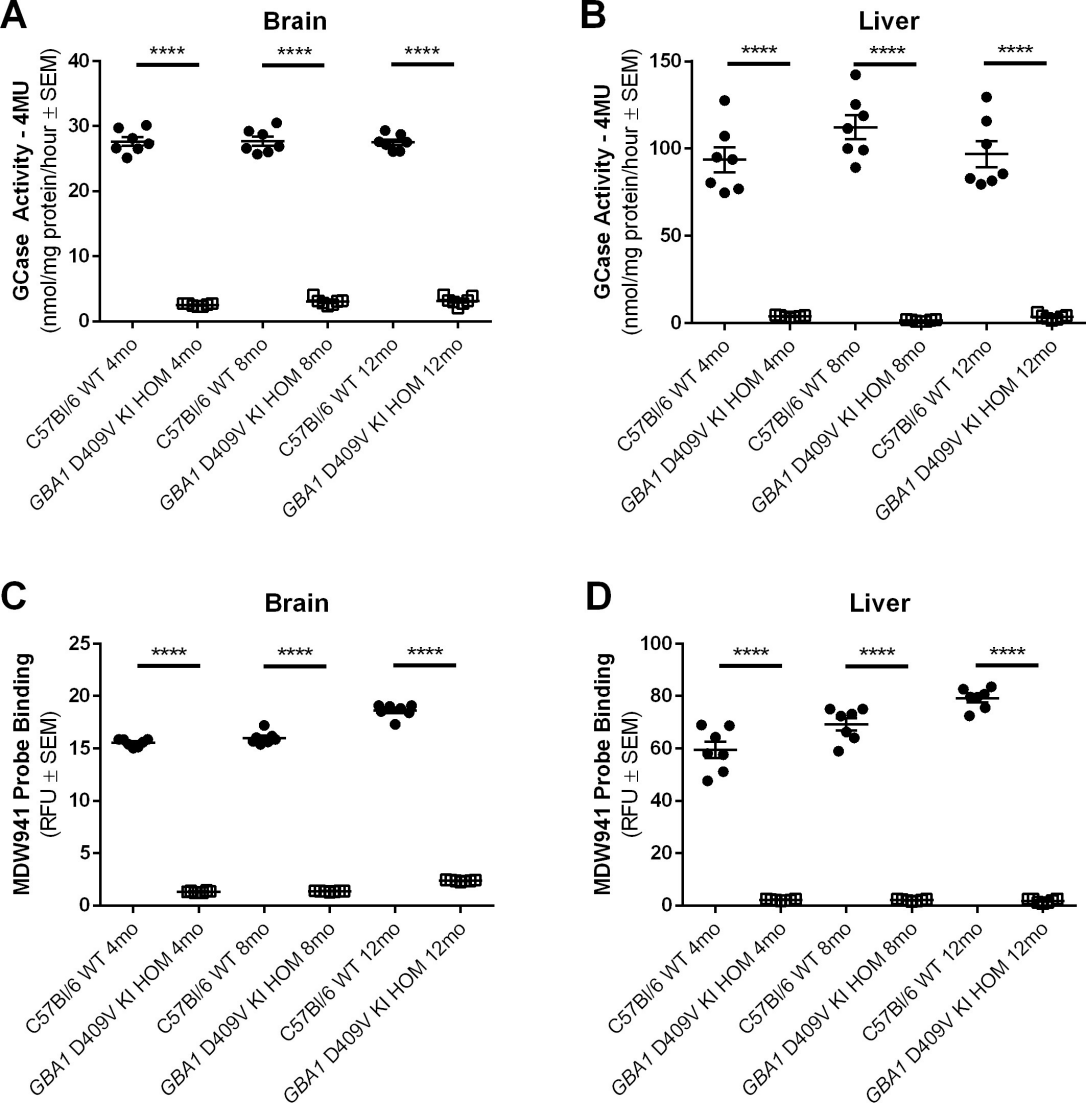
GCase activity is significantly decreased in the brain and liver of the homozygous *GBA1* D409V KI mouse model at 4, 8, and 12 months of age. Two independent groups evaluated GCase activity in whole brain homogenate (A,C) and liver homogenate (B,D) using different methods—The CBE/4-MU method (A,B) and the MDW941 method (C,D). In whole brain homogenate (A,C) and liver homogenate (B,D), GCase activity is significantly decreased in the *GBA1* D409V KI homozygous (HOM) mice as compared to C57Bl/6 wild type (WT) mice (n = 7/group). GCase activity measured by 4-MU in brain (A) measured as follows: WT 4mo 27.63±0.68, HOM 4mo 2.53±0.07, WT 8mo 27.65±0.69, HOM 8mo 3.01±0.20, WT 12 mo 27.49±0.46, HOM 12mo 3.15±0.26. GCase activity measured by 4-MU in liver (B) measured as follows: WT 4mo 93.69±7.16, HOM 4mo 3.90±0.21, WT 8mo 112.40±6.84, HOM 8mo 1.50±0.19, WT 12 mo 96.79±7.44, HOM 12mo 3.50±0.58. GCase activity measured by MDW941 in brain (C) measured as follows: WT 4mo 15.54±0.13, HOM 4mo 1.33±0.04, WT 8mo 15.99±0.23, HOM 8mo 1.36±0.02, WT 12 mo 18.61±0.24, HOM 12mo 2.37±0.02. GCase activity measured by MDW941 in liver (D) measured as follows: WT 4mo 59.48±3.14, HOM 4mo 2.15±0.10, WT 8mo 69.27±2.36, HOM 8mo 2.03±0.13, WT 12 mo 79.14±1.48, HOM 12mo 1.79±0.29. Significant differences are indicated as follows: ****p < 0.0001. Abbreviations: SEM, standard error of the mean; RFU, relative fluorescence units; *GBA1*, gene encoding human glucocerebrosidase; GCase, glucocerebrosidase; WT, wild type; HOM, homozygous; KI, knockin; mo, month.

Our rigorous determination of GCase activity in the *GBA1* D409V KI mouse model utilized brain and liver tissue samples harvested from the same contemporaneous cohorts of mice homozygous for the *GBA1* D409V mutation and evaluated independently by teams at Amicus Therapeutics and Pfizer. At Amicus, lysosomal GCase activity was measured by the CBE-inhibitable release of 4-MU from 4-MUG in buffer (reported as GCase 4MU; **Fig 3A and 3B**). At all ages examined (4, 8, and 12 months) hom *GBA1* D409V KI mice had dramatically reduced GCase activity in brain (main effect of genotype: F(1,36) = 4357, *p* < 0.0001; **Fig 3A**) and nearly negligible GCase activity in liver compared to WT littermates (main effect of genotype: F(1,36) = 561.9, *p* < 0.0001; **Fig 3B**), demonstrating a significant effect of genotype on GCase activity at all ages in all tissues (*p* < 0.0001).

A similarly robust reduction in levels of active GCase in brain and liver of hom *GBA1* D409V KI mice relative to WT controls was observed at Pfizer using the MDW941 activity-based probe method in tissue homogenates from brain and liver samples obtained from the same mice analyzed by Amicus utilizing the CBE/4-MUG method (**Fig 3C and 3D**). MDW941 is an irreversible inhibitor of GCase, 8-deoxy-8-azidocyclophellitol (KY170), bound to a fluorescent molecule (BODIPY). This cell-permeable probe binds with a high degree of selectivity to enzymatically-active GCase molecules in the lysosomal compartment of cells [40, 41] and reflects properly trafficked, localized, and enzymatically-active GCase. Similar to observations using the CBE/4-MUG analytic method at Amicus, GCase activity as measured by the MDW941 probe method was dramatically reduced in brain in hom *GBA1* D409V KI mice at all ages relative to WT controls (main effect of genotype: F(1,35) = 15015, *p* < 0.0001; **Fig 3C**) and was almost completely ablated in liver (main effect of genotype: F(1,36) = 2303, *p* < 0.0001; **Fig 3D**).

Interestingly, in addition to a strong genotype effect, we observed a mild but statistically significant age effect on GCase activity in the MDW941 probe data set. In brain, GCase activity as measured by the MDW941 probe assay was statistically higher in the 12 month old cohort relative to cohorts at both 4 and 8 months old (main effect of age: F(2,35) = 113.0, *p* < 0.0001; WT and *GBA1* D409V KI 4 and 8 vs 12 month comparisons: *p* < 0.0001 for WT, *p* < 0.001 for *GBA1* D409V KI; **Fig 3C**). This apparent age effect of increased GCase activity in brain is seemingly independent of genotype as it applied to both WT and *GBA1* D409V KI cohorts at 12 months of age. In liver samples however, increased GCase activity by higher MDW941 probe readout was evident only in WT mice and was significantly different across all 3 age groups (main effect of age: F(2,36) = 15.76, *p* < 0.0001; WT individual age *post hoc* comparisons: 4 vs 8 and 8 vs 12 months *p* < 0.01, 4 vs 12 months: *p* < 0.001; *GBA1* D409V KI individual age *post hoc* comparisons: *p* > 0.05), demonstrating an apparent correlation between increased GCase activity (determined by higher MDW941 probe signal) with advanced age in WT mice or an inability to detect differences in the different age groups in hom *GBA1* D409V KI mice (**Fig 3D**).

### Glycosphingolipid substrates of GCase are increased in brain and liver from *GBA1* D409V KI mice

GCase is an important enzyme in lysosomal glycolipid metabolism. Deficiency in GCase activity leads to accumulation of specific GSL substrates that have been pathologically attributed to GD [42]. Dysregulated GCase activity and elevated GSLs, such as GlcCer and GlcSph, have been reported in aged humans and PD patients [43] as well as in another *GBA1* D409V mouse model [17, 18, 25] and in non-mutant aged mice [44]. Previous data using a different *GBA1* D409V mouse model shows GCase activity and substrate accumulation to be divergent between peripheral tissues compared to brain [17, 25]. In these studies, GCase activity reduction was more pronounced in the peripheral tissues than in the brain, with a similar pattern observed with regards to substrate accumulation being more pronounced in the periphery as compared to the brain.

**Fig 4:**
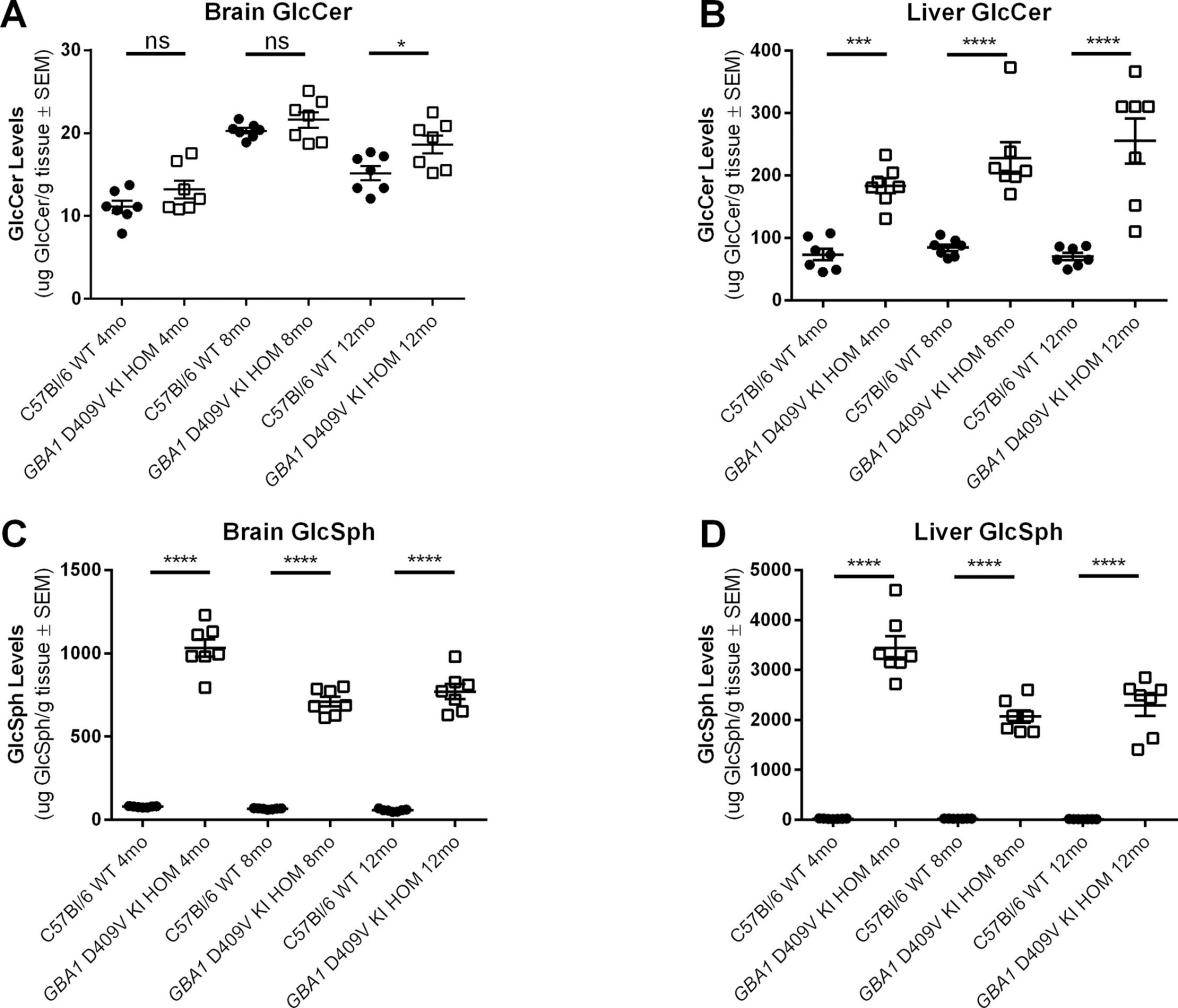
GCase substrate levels are altered in the brain and liver of homozygous *GBA1* D409V KI mice. (A-B) Glucosylceramide (GlcCer) and (C-D) glucosylsphingosine (GlcSph) levels measured by LC-MS/MS in C57Bl/6 wild type (WT) and *GBA1* D409V KI homozygous (HOM) mice at 4, 8, and 12 months of age (n = 7/group). (A) GlcCer levels in the brain are increased in *GBA1* D409V KI mice at 12, but not 4 and 8, months of age. (B) GlcCer levels in the liver are increased in *GBA1* D409V KI mice at all ages as compared to WT mice. (C) GlcSph levels in the brain are significantly increased in *GBA1* D409V KI mice as compared to WT mice at all ages. (D) GlcSph levels in the liver are significantly increased in *GBA1* D409V KI mice at all ages as compared to WT mice. Brain GlcCer (A) measured as follows: WT 4mo 11.20±0.72, HOM 4mo 13.20±1.06, WT 8mo 20.30±0.34, HOM 8mo 21.60±0.95, WT 12 mo 15.17±0.84, HOM 12mo 18.64±1.09. Liver GlcCer (B) measured as follows: WT 4mo 73.56±9.39, HOM 4mo 183.60±12.09, WT 8mo 84.40±5.22, HOM 8mo 228.10±25.32, WT 12 mo 70.30±5.72, HOM 12mo 255.40±35.80. Brain GlcSph(C) measured as follows: WT 4mo 78.98±1.31, HOM 4mo 1032.00±52.91, WT 8mo 66.64±1.38, HOM 8mo 710.30±29.01, WT 12 mo 57.24±2.73, HOM 12mo 770.70±44.97. Liver GlcSph (D) measured as follows: WT 4mo 23.41±2.07, HOM 4mo 3446.00±232.40, WT 8mo 24.47±0.72, HOM 8mo 2071.00±122.30, WT 12 mo 14.73±0.78, HOM 12mo 2291.00±206.50. Significant differences are indicated as follows: *p < 0.05, ***p < 0.001; ****p < 0.0001. Abbreviations: GlcCer, glucosylceramide; GlcSph, glucosylsphingosine; SEM, standard error of the mean; *GBA1*, gene encoding human glucocerebrosidase; GCase, glucocerebrosidase; WT, wild type; HOM, homozygous; KI, knockin; mo, month; ns, non-significant (*p* > 0.05).

To assess the functional consequences of decreased GCase activity in our *GBA1* D409V KI mouse model, GSLs downstream of GCase were evaluated by the teams at Amicus and Pfizer in the same brain and liver samples from hom *GBA1* D409V KI mice that were used to measure GCase activity. In liver, GlcCer was significantly higher in hom *GBA1* D409V KI mice compared to GlcCer levels in WT mice at all three ages examined (main effect of genotype: F(1,36) = 86.88, *p* < 0.0001; 4 month WT vs *GBA1* D409V KI: *p* < 0.01; 8 and 12 month WT vs *GBA1* D409V KI: *p* < 0.0001; **Fig 4B**). The effects of decreased GCase activity on GlcCer levels in brain were also significant (main effect of genotype: F(1,36) = 10.33, *p* < 0.01) but more subtle as only the 12 month old cohort of hom *GBA1* D409V KI mice exhibited significantly elevated levels of GlcCer compared to WT controls (*p* < 0.05; **Fig 4A**). Trends toward increased GlcCer in brain tissue from hom *GBA1* D409V KI mice at 4 and 8 months of age relative to WT did not reach statistical significance (*p* > 0.05; **Fig 4A**). Relatedly, levels of GlcSph, another important GSL substrate of GCase enzymatic activity, were dramatically increased in liver (main effect of genotype: F(1,36) = 537.6, *p* < 0.0001; **Fig 4D**) and brain (main effect of genotype: F(1,36) = 940.7, *p* < 0.0001; **Fig 4C**) in hom *GBA1* D409V KI mice compared to matched WT controls at all ages (*p* < 0.0001).

Moreover, a potential age effect for GlcCer in brain (main effect of age: F(2,36) = 51.04, *p* < 0.0001; **Fig 4A**) but not liver (main effect of age: F(2,36) = 1.794, *p* = 0.1808; **Fig 4B**) was observed. In brain, GlcCer levels appear to vary with age in both WT (4 versus 8 months: *p* < 0.0001; 8 versus 12 months: *p* < 0.01; 4 versus 12 months: *p* < 0.05) and hom *GBA1* D409V KI mice (4 versus 8 months: *p* < 0.0001; 4 versus 12 months: *p* < 0.01; 8 versus 12 months: *p* > 0.05). Age-related changes in GlcSph levels in brain and liver were also assessed. There were significant differences in GlcSph levels in brain (main effect of age: F(2,36) = 17.15, *p* < 0.0001; **Fig 4C**) and liver (main effect of age: F(2,36) = 14.70, *p* < 0.0001; **Fig 4D**). In both regions, however, the age-related changes appear to be restricted to the *GBA1* D409V KI mice and early age (4 versus 8 months: *p* < 0.0001; 4 versus 12 months: *p* < 0.0001; 8 versus 12 months: *p* > 0.05). Importantly, the aged mouse cohorts of hom *GBA1* D409V KI and WT mice were not longitudinally assessed, so reported age-related differences refer to distinctions between the means of individual age-matched cohorts of WT and hom *GBA1* D409V KI mice compared to other genetic cohorts at different ages.

### Intermediate decrease of GCase enzyme activity and increased glycosphingolipid substrates in brain and liver from heterozygous *GBA1* D409V KI mice

**Fig 5:**
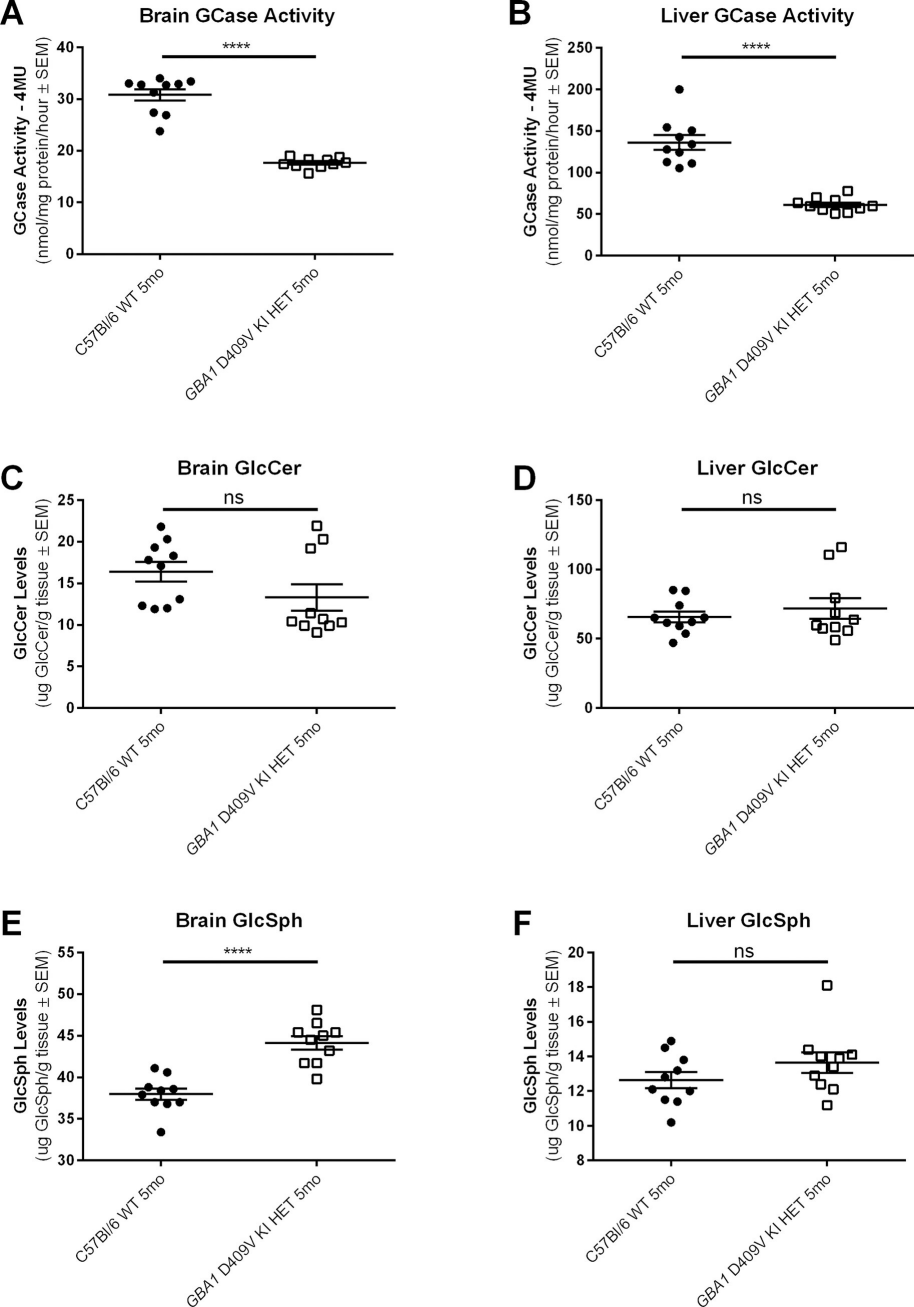
Brain and liver GCase activity are significantly decreased in heterozygous *GBA1* D409V KI mice, with varying differences in glucosphingolipid lipids in brain and liver. (A-B) GCase, (C-D) gluocosylceramide (GlcCer), and (E-F) glucosylsphingosine (GlcSph) levels measured in whole brain homogenate and liver homogenate of C57Bl/6 wild type (WT) and *GBA1* D409V KI heterozygous (HET) mice at 5 months of age (n = 10/age). (A-B) GCase levels as assessed by the CBE/4-MU method are significantly decreased in the het *GBA1* D409V KI mice at 5 months of age in both brain (A) and liver (B). (C-D) GlcCer levels in the brain (C) and liver (D) are unchanged in het *GBA1* D409V KI mice as compared to WT mice at 5 months of age. (E-F) GlcSph levels are significantly increased in het *GBA1* D409V KI mice as compared to WT mice in brain (E) but not liver (F). GCase activity measured by 4-MU in brain (A) measured as follows: WT 30.82±1.11, HET 17.65±0.32. GCase activity measured by 4-MU in liver (B) measured as follows: WT 136.20±8.82, HET 61.20±2.69. Brain GlcCer (C) measured as follows: WT 16.39±1.18, HET 13.31±1.59. Liver GlcCer (D) measured as follows: WT 65.80±3.90, HET 71.88±7.37. Brain GlcSph (E) measured as follows: WT 37.96±0.68, HET 44.13±0.80. Liver GlcSph (F) measured as follows: WT 12.64±0.47, HET 13.65±0.59. Significant differences are indicated as follows: ****p *< 0*.*0001*. Abbreviations: GlcCer, glucosylceramide; GlcSph, glucosylsphingosine; *GBA1*, gene encoding human glucocerebrosidase; GCase, glucocerebrosidase; WT, wild type; HET, heterozygous; KI, knockin; SEM, standard error of the mean; mo, month; ns, non-significant (*p* > 0.05).

To investigate potential gene dosing effects of *GBA1* D409V KI and because PD patients with mutations in the *GBA1* gene tend to be heterozygous, we also examined GCase activity and GSL levels in mice heterozygous for the *GBA1* D409V KI mutation compared to WT mice, using the same methodology deployed at Amicus for analyses of hom *GBA1* D409V KI mice. GCase activity (using the CBE/4-MUG method) was found to be decreased in 5 month old het *GBA1* D409V KI mice compared to age-matched WT controls but to an intermediate extent relative to mice homozygous for the *GBA1* D409V KI mutation. Specifically, GCase activity was decreased in both brain (*p* < 0.0001; **Fig 5A**) and liver (*p* < 0.0001; **Fig 5B**) in 5 month old het *GBA1* D409V KI mice compared to WT control mice at the same age, but not to the same extent as the observed decrease in GCase activity in brain and liver of hom *GBA1* D409V KI mice at 4, 8, and 12 months of age (compare **Fig 5A and 5B** to **Fig 3**). Levels of GlcCer and GlcSph were also examined in the 5 month old het *GBA1* D409V KI mice. No statistically significant genotype-related differences in GlcCer were observed in het *GBA1* D409V KI mice relative to WT in either brain (*p* = 0.0658; **Fig 5C**) or liver (*p* > 0.9999; **Fig 5D**). However, a significant increase in GlcSph in brain in 5 month old het *GBA1* D409V KI mice compared to age-matched WT control mice was noted (*p* < 0.0001; **Fig 5E**), while levels of GlcSph in liver were not significantly different in het *GBA1* D409V KI mice compared to WT controls (*p* = 0.1967; **Fig 5F**).

### Lysosomal Lamp1 protein levels in brain and liver are unchanged in the *GBA1* D409V KI mouse

Deficiency in GCase activity in GD leads to stereotyped physiological effects, including hepatosplenomegaly due to lysosomal engorgement because of accumulated GSL substrates and heightened inflammation from activation of tissue-resident macrophages, referred to as Gaucher cells [45]. In addition to dysregulation of the autophagic-lysosomal pathway in GD, alterations in this system have also been reported in PD [46]. Lysosome-associated protein 1 (Lamp1), is highly enriched in the lysosomal membrane and is integral for lysosomal biogenesis and autophagy [47]. Thus, Lamp1 protein expression can provide a proxy measurement of general lysosomal integrity. Previously, knocked-down GCase was associated with increased Lamp1 expression and diminished clearance of alpha-synuclein in neurons [9].

Thus, we next sought to determine if lysosomal function was globally affected as a result of KI of *GBA1* D409V in our mouse model. Lysosomal levels of Lamp1 were analyzed at Pfizer in brain and liver tissue homogenate from the same cohorts of age-matched hom *GBA1* D409V KI and WT mice that were evaluated for GCase activity (**Fig 3C and 3D**) and GSL levels. No significantly different changes in levels of Lamp1 were observed comparing *GBA1* D409V KI mice to WT controls at any age examined in either brain (main effect of genotype: F(1,36) = 1.419, *p* = 0.2414; **S1A Fig**) or liver tissue (main effect of genotype: F(1,36) = 0.5758, *p* = 0.4529; **S1B Fig**). However, age-related changes in Lamp1 protein were observed in brain (main effect of age: F(2,36) = 19.22, *p* < 0.0001; **S1A Fig**) and liver (main effect of age: F(2,36) = 94.62, *p* < 0.0001; **S1B Fig**). In brains of *GBA1* D409V KI mice, levels of lysosomal Lamp1 by immunoblot were higher in 12 month old as compared to 4 month (*p* < 0.01) and 8 month (*p* < 0.01) mice, but not for 8 month old compared to 4 month old (*p* > 0.05) (**S1A Fig**). Brain levels of Lamp1 in WT mice were lower in 4 month old WT mice compared to 12 month old WT mice (*p* < 0.01), but no statistically significant differences in Lamp1 levels were observed between any other WT aged mice (*p* > 0.05; **S1A Fig**).

In liver, Lamp1 levels were greater in 12 month old hom *GBA1* D409V KI mice compared to both the 4 month (*p* < 0.0001) and 8 month old (*p* < 0.0001) hom cohorts; no apparent age-related differences were observed comparing the 4 and 8 month old hom *GBA1* D409V KI groups (*p* > 0.05; **S1B Fig**). Similar to the *GBA1* D409V KI mice, WT mice demonstrated elevated Lamp1 levels at 12 months of age as compared to 4 months (*p* < 0.0001) and 8 months (*p* < 0.001), with no apparent age-related differences between 4 and 8 months (*p* > 0.05; **S1B Fig**).

### Dopamine neurons in the substantia nigra remain intact in *GBA1* D409V KI mice

**Fig 6:**
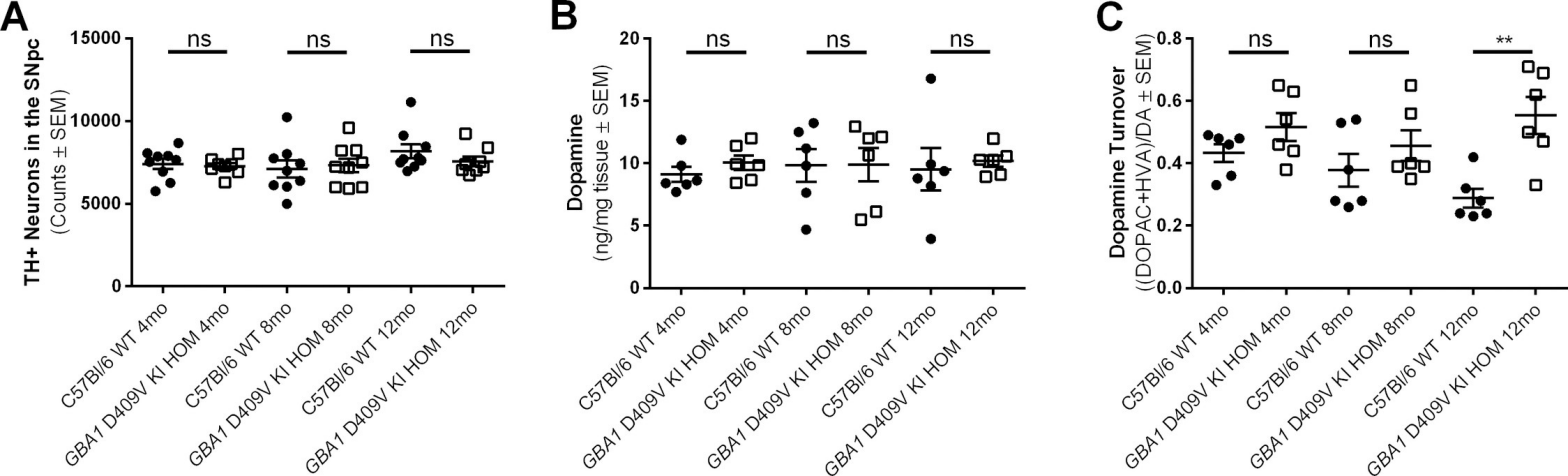
Dopamine turnover, but not total dopamine levels or nigral dopamine neuron numbers, are altered in aged homozygous *GBA1* D409V KI mice as compared to controls. (A) Stereological estimates of dopaminergic neurons in the substantia nigra pars compacta (SNpc) as denoted by tyrosine hydroxylase immunoreactivity (TH+) reveal no differences between the *GBA1* D409V KI homozygous (HOM) mice and C57Bl/6 wild type (WT) mice at any age (n = 9/group). (B) Dopamine levels as assessed by UHPLC/MS/MS in striatal tissue are unaltered in HOM *GBA1* D409V KI mice at all ages (n = 6/group). (C) Dopamine turnover is significantly increased in 12 month old HOM *GBA1* D409V KI mice as compared to WT controls (n = 6/group). TH neurons (A) measured as follows: WT 4mo 7402±306.7, HOM 4mo 7263±181.9, WT 8mo 7102±503.5, HOM 8mo 7319±412.9, WT 12 mo 8169±429.1, HOM 12mo 7566±294.9. Dopamine levels (B) measured as follows: WT 4mo 9.13±0.62, HOM 4mo 10.05±0.58, WT 8mo 9.84±1.31, HOM 8mo 9.89±1.33, WT 12 mo 9.50±1.70, HOM 12mo 10.18±0.45. Dopamine turnover (C) measured as follows: WT 4mo 0.43±0.03, HOM 4mo 0.52±0.04, WT 8mo 0.38±0.05, HOM 8mo 0.46±0.05, WT 12 mo 0.29±0.03, HOM 12mo 0.55±0.06. Significant differences are indicated as follows: ***p < 0.001. Abbreviations: TH+, tyrosine hydroxylase immunoreactive; SNpc, substantia nigra pars compacta; DOPAC, 3,4-dihydroxyphenylacetic acid; HVA, homovanillic acid; DA, dopamine; *GBA1*, gene encoding human glucocerebrosidase; WT, wild type; HOM, homozygous; KI, knockin; SEM, standard error of the mean; mo, month; ns, non-significant (*p* > 0.05).

The histopathological hallmark of PD is the death of dopaminergic neurons in the substantia nigra of the ventral midbrain. However, overt loss of nigral DA neurons is not often observed in genetic mouse models of parkinsonism. We deployed the unbiased stereological method of cell number estimation to quantify the number of DA neurons in the SNpc of our hom *GBA1* D409V KI mice at 4, 8, and 12 months of age compared to age-matched WT controls. No statistically significant effects on number of dopamine neurons were noted for either genotype or age (main effect of genotype: F(1,46) = 0.3200, *p* = 0.5743; main effect of age: F(2,46) = 1.692, *p* = 0.1954; **Fig 6A**).

### Dopamine turnover is increased in aged *GBA1* D409V KI mice

Dysregulated DA neuron cellular function results in the loss of DA tone in the striatum, causing the stereotypical motor deficits observed in PD. Thus, changes in DA neurotransmitter levels as well as DA metabolites and other neurotransmitters occurs in PD patients and in some preclinical disease models. Increased DA turnover is reported to be an early occurrence in PD and relates to disease-compensatory mechanisms [48].

Using a published HPLC/MS/MS technique, we quantified DA, its metabolites, and its turnover in the striatum of WT and hom *GBA1* D409V KI genotyped mice at 4, 8, and 12 months of age. No statistically significant effects for either genotype or age were observed for DA levels (main effect of genotype: F(1,30) = 0.3700, *p* = 0.5476; main effect of age: F(2,30) = 0.0384, *p* = 0.9624; **Fig 6B**). Notably though, we did observe a genotype effect for DA turnover (main effect of genotype: F(1,30) = 14.72, *p* < 0.001); at 12 months of age, hom *GBA1* D409V KI mice had significantly increased DA turnover relative to WT mice at the same age (*p* < 0.01; **Fig 6C**). Trends toward increased DA turnover in hom *GBA1* D409V KI mice at 4 and 8 mo relative to WT controls at the same ages did not reach significance. Moreover, age did not appear to impact DA turnover as it was equivalent within the respective genotypes across all ages examined (main effect of age: F(2,30) = 1.011, *p* = 0.3760; **Fig 6C**).

### *GBA1* D409V KI mice lack alpha-synuclein pathology and neuroinflammation in the striatum and midbrain

Alpha-synuclein has been genetically and pathologically linked to PD [2]. In addition, GCase has been shown to be important in regulating the turnover of aSyn—presumably via the autophagy-lysosome system—with mutant *GBA1* implicated in exacerbating synucleinopathy in some, but not all, models [18, 19, 21, 49–51]. We therefore sought to assess levels of total aSyn and its phosphorylated form, pS129 (as a marker of pathologic aSyn) by immunohistochemistry (IHC) in the brains of *GBA1* D409V KI mice. We did not observe obvious differences in either total or pS129 aSyn by IHC at the level of striatum or SN in the brains of hom *GBA1* D409V KI mice relative to WT controls at 12 months of age (**S2 Fig**).

CNS inflammation is a hallmark of GD and has also been observed in the context of heterozygous mutations in *GBA1* [52, 53]. Moreover, neuroinflammation is also a pathologic feature of PD [54] and has been reported in the hippocampus of one study utilizing this *GBA1* D409V model [30] but not in another *GBA1* D409V mouse model [18]. Thus, we examined microgliosis and astrogliosis in the striatum and substantia nigra of our *GBA1* D409V KI mouse model. Although an age-related increase in microglial reactivity and astrogliosis was observed in the striatum and substantia nigra, no differences between WT and *GBA1* D409V KI mice were apparent at any age (**S3 Fig**).

## Discussion

While there are numerous genetic and environmental factors associated with the complex etiology of PD, the exact cause of most cases of PD is unknown. It is presumed, however, that many of the causes of PD converge on conserved mechanistic pathways that contribute to pathology and manifestation of the disease. One such example of distinct factors converging on common pathways and leading to conserved pathology is that of aSyn and GCase. Dysregulation of aSyn downstream of aberrant GCase activity contributes fundamentally to the proposed hypothesis of aSyn accumulation, aggregation, and possibly prion-like cell-to-cell transmission of pathology, which together contribute to PD initiation and progression [24, 55, 56]. In PD patients harboring *GBA1* mutations, aSyn pathology is present and more pronounced in neocortical regions [28]. Conversely, sporadic PD patients demonstrate reduced GCase in brain tissues [6, 8, 27], indicating a potential therapeutic strategy of augmenting GCase to treat idiopathic PD. To better understand the role of decreased GCase activity and develop a model for testing potential therapeutic candidates targeting GCase in genetic and idiopathic PD cases, we set out to develop and evaluate a novel preclinical *GBA1* D409V KI mouse model. The *GBA1* D409V KI mice are viable and exhibit no overt developmental or neurological deficits. Upon deeper molecular, neurochemical, histological, and behavioral analyses, the *GBA1* D409V KI model displays relevant outcomes useful for modeling GCase deficiency and is consistent with features reported in other GCase-deficient models (**Table 1**) [30, 31].

**Table 1:** Characterization summary the *GBA1* D409V KI mouse (JAX Strain 019106).

**GCase Activity**						
	GBA1 mRNA	GCase Protein	GCase Activity	GlcCer Levels	GlcSph Levels	Lysosome Function
Reported Herein	**HOM Brain (4mo):** Unchanged	**HOM Brain (4mo):** Unchanged	**HOM Brain (4,8,12mo):** ~90% Decrease	**HOM Brain (4,8,12mo):**Unchanged	**HOM Brain (4,8,12mo):** 10.5–13.5x Increase	**HOM Brain (4,8,12mo):** Unchanged
			**HET Brain (5mo):** ~45% Decrease		**HET Brain (5mo):** 1.2x Increase	
				**HET Brain (5mo):** Unchanged		
					**HOM Liver (4,8,12mo):** 85-155x Increase	
				**HOM Liver (4,8,12mo):** 2.5–3.5x Increase		
			**HOM Liver (4,8,12mo):** ~95% Decrease			
	**HET Brain (4mo):** Unchanged				**HET Liver (5mo):** Unchanged	
				**HET Liver (5mo):** Unchanged		
						**HOM Liver (4,8,12mo):** Unchanged
		**HET Brain (4mo):** Unchanged				
			**HET Liver (5mo):** ~55% Decrease			
[30]	Not Assessed	Not Assessed	**GBA1 HOM HPC/CTX (12mo):** ~95% Decrease	Not Assessed	Not Assessed	Not Assessed
			**GBA1 HET HPC/CTX (12mo):** ~70% Decrease			
			**GBA2 HOM HPC (12mo):** ~90% Decrease			
			**GBA2 HET HPC (12mo):** ~65% Decrease			
			**GBA2 HOM/HET CTX (12mo):** Unchanged			
[31]	Not Assessed	Not Assessed	**HET HPC (13-15mo):** ~20% Decrease	**HET HPC (13-15mo):** Unchanged	**HET HPC (13-15mo):** Unchanged	Not Assessed
**PD-Related Motor Phenotypes and Pathology**						
	Motor Behavior	STR Neurochemistry	SN DA Neurons Numbers	STR/SN Inflammation	STR/SN aSyn Expression	STR/SN aSyn Pathology
Reported Herein	**HOM (4,8,12 mo):** Absent	**HOM (4,8 mo):** Unchanged	**HOM (4,8,12 mo):** Unchanged	**Astrocytes in HOM (12 mo):** Unchanged	**HOM (4,8,12 mo):** Unchanged	**HOM (4,8,12 mo):** Absent
		**HOM (12 mo):** 2x DA Turnover Increase		**Microglia in HOM (12 mo):** Unchanged		
[30]	**HET (12 mo):** Absent	Not Assessed	Not Assessed	Not Assessed	Not Assessed	Not Assessed
[31]	Not Assessed	Not Assessed	**HET (13-15mo):** Unchanged	Not Assessed	Not Assessed	Not Assessed

Abbreviations: HOM, homozygous; HET, heterozygous; mo, month; GlcCer, glucosylceramide; GlcSph, glucosylsphingosine; HPC, hippocampus; CTX, cortex; STR, striatum; SN, substantia nigra; DA, dopamine; aSyn, alpha-synuclein

Specifically, hom *GBA1* D409V KI mice have dramatically decreased GCase activity as measured in multiple target organs and by multiple methods (**Fig 3**). These results are consistent with another study reporting substantial loss in GCase activity in other brain regions of this model (**Table 1**) [30]. Modest loss of GCase activity was also reported in another *GBA1* D409V KI mouse model (**Table 2**) [17]; differences in the magnitude of the loss are most likely attributed to differences in genetic background of the mouse models [57]. This effect was not the result of changes in transcription/translation as the *GBA1* D409V KI mutation did not appear to affect total *GBA1* mRNA or GCase protein levels was compared to WT mice (**Fig 2**). The decrease in GCase activity in liver in hom *GBA1* D409V KI mice correlated with elevated total GlcCer and GlcSph in hom *GBA1* D409V KI mice compared to WT controls (**Fig 4**). In brain specimens, deficits in GCase activity correlated with robust elevations across all age groups of GlcSph, but not GlcCer. These findings are consistent with brain GlcCer and GlcSph levels reported across multiple GBA mutant mouse models [58]. Deacylation of GlcCer to GlcSph by lysosomal acid ceramidase has been suggested to occur as a metabolic adaptation to reduced GCase activity [59], and as such, GlcSph had emerged as a sensitive and selective biomarker of GBA-related disorders [60].

**Table 2:** Phenotype comparisons between different homozygous *GBA1* D409V KI mouse models.

	MJFF *GBA1* D409V KI Mouse (Reported Herein)	Grabowski *GBA1* D409V KI Mouse
Background Strain	C57Bl/6	Mixed C57Bl/6 and 129/SvEvBrd [17]
Lifespan	Unaffected by mutation	Unaffected by mutation [17]
*GBA1* mRNA	100% of WT	50–100% of WT [17]
GCase Activity in Brain	~10% of WT	22.5–25% of WT [17, 18]
GCase Activity in Liver	~5% of WT	~2.5% of WT [17]
GlcCer in Brain	100% of WT	100% of WT [17]
GlcCer in Liver	2.5–3.5 fold increase vs WT	2–4 fold increase vs WT [17, 18]
GlcSph in Brain	10.5–13.5 fold increase vs WT	3–5 fold increase vs WT [18]
SNpc Cell Loss	Unaffected by mutation	Unaffected by mutation [18]
Lysosomal Function	Unaffected by mutation	Unaffected by mutation [18]
aSyn Pathology	Unaffected by mutation in STR/SN and increased in HPC [30]	Unaffected by mutation [21, 49] or increased by mutation in HPC [18, 51]
Inflammation	Unaffected by mutation in STR/SN and increased in HPC [30]	Unaffected by mutation in HPC [18]
Motor Phenotypes	Unaffected by mutation	Unaffected by mutation [18]

Abbreviations: KI, knockin; GlcCer, glucosylceramide; GlcSph, glucosylsphingosine; HPC, hippocampus; STR, striatum; SNpc, substantia nigra pars compacta

Notably, we also found that mice heterozygous for the *GBA1* D409V KI mutation exhibited a significant decrease in GCase activity in both liver and brain, albeit not at the same magnitude as hom *GBA1* D409V KI mice (**Figs 3 and 5**). Effects of the het *GBA1* D409V KI were also more subtle on GSL levels as compared to hom *GBA1* D409V KI mice (**Figs 4 and 5**). In contrast to the consistent elevation in GlcSph levels in hom *GBA1* KI mice across ages and tissues, GlcSph was increased only in brain tissue in het *GBA1* D409V KI mice. The levels of GlcCer in het *GBA1* D409V KI mice were unchanged in both brain and liver as compared to WT mice. These results are largely consistent with another report of this model by Burbulla et al (2019) who found hippocampal levels of GlcCer unchanged and only a slight trend towards increased GlcSph in het mice at 13–15 months of age (**Table 1**) [31].

Additionally, the biological significance of an apparent age effect of increased GCase activity in the MDW941 data set warrants further investigation. A biologically meaningful age-related increase in GCase activity is not certain, as it was observed by only one method (MDW941 activity probe and not by the CBE/4-MU method) and diverged between organs (brain versus liver) and genotype (WT versus *GBA1* D409V KI) (**Fig 3C and 3D**). One possible explanation for the divergent results of increased GCase activity in *GBA1* D409V KI brain but not liver samples could be due to a floor effect in the *GBA1* D409V KI liver samples, whereby GCase activity in liver was already negligible at 4 months of age and could not measurably or functionally decrease any further. It is also possible that assessment of GCase activity by the MDW941 probe method is more sensitive than the CBE/4-MU method, as an age-related increase in GCase activity was not detected by both methods using the same tissue samples.

Global age-related decreases in GCase and accumulation in GSLs—specifically GlcCer and GlcSph—has been reported in other published studies as a consequence of normal aging [44]. While this diverges with our observation that GCase activity was unchanged with age in WT mice using the CBE/4-MU method and increased in brain using the MDW941 probe method, these differing observations could be attributable to fundamental distinctions between the studies for experimental design/analyses, mouse strains examined, and different ages of the mice assessed. Additional research into age-related changes in GCase activity and GSL accumulation in different inbred mouse strains using a variety of methods would be warranted to determine if different genetic backgrounds impact basal GCase activity or GSL accumulation across the lifespan and how this may relate to any resulting pathology like that of the aSyn protein [44, 60].

While we observed multiple GCase-specific phenotypic effects as a consequence of the *GBA1* D409V KI mutation at the biochemical level in both the periphery and the CNS, we did not observe Gaucher cells in any of the tissues examined histologically (including brain, liver and spleen). Moreover, other CNS-related PD-relevant readouts likewise showed no genotype effect (**Fig 6**). Specifically, stereological quantification of DA neurons in the SNpc yielded similar cell number estimates between hom *GBA1* D409V KI mice and WT controls at all ages examined. This finding was also reported in studies of other *GBA1* D409V mouse models (**Table 2**) [18]. Consistent with the lack of overt nigral neuron loss, the hom *GBA1* D409V KI mice did not exhibit changes in striatal DA levels as compared to WT mice. The hom *GBA1* D409V KI mice did, however, display a slight decrease in DA turnover at 12 months of age. This finding is intriguing given that striatal DA turnover is regarded as an early, more subtle perturbation of the nigrostriatal system [48]. The extent to which the nigrostriatal system is impaired in this model, at least up to 12 months of age, is minimal and does not lead to overt motor behavior deficits in the hom *GBA1* D409V KI mice as assessed in this study by open field, accelerating rotarod, fore- and hindlimb grip strength, and posture/gait and in het *GBA1* D409V KI mice as assessed by rotarod and open field testing [30]. This finding is consistent with another *GBA1* D409V mouse model [18] and the existing body of literature indicating that motor deficits are typically not observed in preclinical animal models of parkinsonism unless there is significant nigrostriatal degeneration [61, 62].

Although we did not assess behavioral outcomes associated with learning or spatial memory, these phenotypes have been reported in this and other *GBA1* D409V KI mouse models previously [18, 30]. Deficits in Y-maze performance and the Morris water maze were present in this *GBA1* D409V KI mouse model at 12 months of age in heterozygous mutation carriers. Importantly, these functional deficits corresponded to alterations in hippocampal neurochemistry and inflammation, but not synuclein pathology in the hippocampus [30]. As pathogenic *GBA1* mutations are associated with early and more advanced cognitive decline in PD patients [63], the demonstration of this phenotype in this and other *GBA1* D409V KI mouse models [18, 30] is of interest.

Somewhat surprisingly given the global, early, and profound decreased GCase activity, we did not observe alterations in aSyn expression in the midbrain or striatum of the hom *GBA1* D409V KI mice (**S2 Fig**) as measured by IHC at the ages studied. Although Clarke *et al* (2019) did report an increase in aSyn expression in the hippocampus of 12 month old hom *GBA1* D409V KI mice [30], the immunoassay employed in that study was more sensitive and quantitative than the IHC method used in this study. As greater pathology was observed in the hippocampus of this model [30] than the nigrostriatal system, these differences could also be due to structure-related differences in the ability to process/clear accumulation of aSyn. Further evidence for this possibility can be found in the lack of changes in lysosomal Lamp1 expression in brain measured by immunoblot (**S1 Fig**). Notably, other groups have reported that loss of GCase activity does not affect lysosomal function in neuronal cells [64], another *GBA1* D409V mouse model (**Table 2**) [18], and in aSyn A53T-overexpressing mice after GCase inhibition with CBE [10].

In addition to lack of aSyn accumulation, we also do not report any increases in aSyn phosphorylation or aggregation in the striatum or SN of the hom *GBA1* D409V KI mice (**S2 Fig**) as measured by IHC up to 12 months of age. Reports of synuclein pathology in *GBA1* D409V mouse models have been inconsistent in the past. In another hom *GBA1* D409V mouse model, two studies failed to show aSyn pathology as assessed by IHC and immunoblot for insoluble aSyn up to 12 months of age [21, 49] while Sardi *et al* (2011, 2013) report an increase in insoluble aSyn aggregates at 6 and 12 months of age as assessed by IHC in this same model [18, 51]. Even in this specific *GBA1* D409V mouse model results have not been consistent. Clarke *et a*l (2019) reported an absence of aSyn pathology in the hippocampus of this *GBA1* D409V mouse line at 12 month old in heterozygous mice using pS129 and PK-resistant aSyn IHC while Burbulla *et al* (2019) did report the presence of triton-insoluble aSyn in the hippocampus of 13–15 month old het *GBA1* D409V KI mice as assessed by immunoblot [30, 31]. The difference in reports of synuclein pathology may be due to the methods used for assessing synuclein pathology and isolation of specific brain regions for synuclein pathology analysis. Thus, a more detailed study of aggregated aSyn—oligomeric or fibrillar—in the hom *GBA1* D409V KI could be informative. Furthermore, as Parkinson’s disease is a highly complex, age-dependent disease spanning many physiological functions, additional environmental factors and/or extended aging may be needed to trigger aSyn pathology and overt PD-like phenotypes in the *GBA1* D409V KI.

Finally, we investigated neuroinflammation in the midbrain and striatum of *GBA1* D409V KI mice due to associations with GBA and neuroinflammation in GD and PD patients and reports of increased microgliosis and astrogliosis in the hippocampus of the *GBA1* D409V KI model at 12 months of age (**Table 1**) [30]. In the striatum and substantia nigra of the *GBA1* D409V KI mouse there were no signs of an increase in microgliosis or astrogliosis as compared to the WT controls at any age (**S3 Fig**). The lack of neuroinflammation we observe here is not unique. Sardi *et al* (2011) also reported absence of inflammation in the hippocampus of another homozygous *GBA1* D409V KI mouse (**Table 2**) [18].

Collectively, the *GBA1* D409V KI model described herein demonstrates early, sustained decreases in GCase activity in the brain and periphery with concomitant increases in GSL substrates of GCase, as well as early manifestations of nigrostriatal dysfunction as demonstrated by decreased DA turnover at 12 months of age (**Table 1**). This model will serve as an important tool for groups looking to test therapeutic interventions aimed at increasing GCase levels or activity, or combating the increased GSL levels resulting from GCase deficiencies. Studies using the *GBA1* D409V KI mouse will hopefully lead to new avenues of research into the role of GCase in DLB or PD and the development of new therapies aimed at the pathology resulting from dysregulation of this protein and its enzymatic function.

## Supporting information

S1 Fig Lysosome function, as assessed by Lamp1 levels, are unchanged in the *GBA1* D409V KI mouse model. (A-B) Lamp1 levels were measured by Western blot in brain and liver homogenate from C57Bl/6 wild type (WT) mice and *GBA1* D409V KI homozygous (HOM) mice at 4, 8, and 12 months of age (n = 7/group). Levels of Lamp1 were not significantly different between WT and *GBA1* D409V KI mice at any age in brain (A) or liver (B) tissue. There is an age-related increase in Lamp1 appearing at 12 months of age, with statistically significant differences in the brain and liver of *GBA1* D409V KI mice and the liver of WT mice. Lamp1 protein levels are normalized to β-actin as the housekeeping protein. Brain LAMP1 (A) measured as follows: WT 4mo 103.6±3.35, HOM 4mo 98.95±4.40, WT 8mo 124.8±3.11, HOM 8mo 130.7±3.40, WT 12 mo 93.27±2.14, HOM 12mo 121.80±1.98. Liver LAMP1 (B) measured as follows: WT 4mo 0.35±0.02, HOM 4mo 0.37±0.02, WT 8mo 0.30±0.02, HOM 8mo 0.32±0.02, WT 12 mo 0.33±0.01, HOM 12mo 0.48±0.04. Abbreviations: LAMP1, lysosomal associated membrane protein 1; *GBA1*, gene encoding glucocerebrosidase; WT, wild type; HOM, homozygous; KI, knockin; SEM, standard error of the mean; mo, month; ns, non-significant (*p* > 0.05).
(TIF)

S2 Fig Total and phosphorylated alpha-synuclein levels are unchanged in the homozygous *GBA1* D409V KI mouse brain at 12 months of age. Representative images of immunohistochemical staining for total alpha-synuclein (aSyn; A-B, E-F) and phosphorylated S129 aSyn (pS129 aSyn; C-D, G-H) in C57Bl/6 wild type (WT; A, C, E, G) and *GBA1* D409V KI homozygous (HOM; B, D, F, H) mice reveal no overt differences in total aSyn or pS129 aSyn levels between genotypes in either the striatum (A-D) or substantia nigra (SN; E-H). Primed images (A’-H’) are higher magnification images taken using the 10x objective lens at the region of interest denoted with an inset box (A-H). Abbreviations: aSyn, alpha-synuclein; pS129, phosphorylated serine 129; SN, substantia nigra; *GBA1*, gene encoding human glucocerebrosidase; WT, wild type; HOM, homozygous; KI, knockin.
(TIF)

S3 Fig Microgliosis and astrogliosis are not exacerbated by homozygous GBA1 D409V mutation in the striatum and substantia nigra. Representative images of immunohistochemical staining for astrocytes using GFAP (green), microglia using Iba-1 (red), dopaminergic neurons using tyrosine hydroxylase (TH; orange), and nuclei using DAPI (blue) in C57Bl/6 wild type (A-B, E-F, I-J) and GBA1 D409V KI homozygous (C-D, G-H, K-L) mice reveal no overt differences in neuroinflammation between genotypes in either the striatum (A, C, E, G, I, K) or substantia nigra (B, D, F, H, J, L). Inset panels are higher magnification images taken using the 40x objective lens. Abbreviations: GBA1, gene encoding human glucocerebrosidase; WT, wild type; KI, knockin.
(TIF)

S1 File Raw data. (DOCX)
