# Supplementary figures and images for: Decreased glucocerebrosidase activity and substrate accumulation of glycosphingolipids in a novel GBA1 D409V knock-in mouse model
Source: PLoS One. 2021 Jun 9;16(6):e0252325. doi: 10.1371/journal.pone.0252325 (PMC8189458; doi:10.1371/journal.pone.0252325)

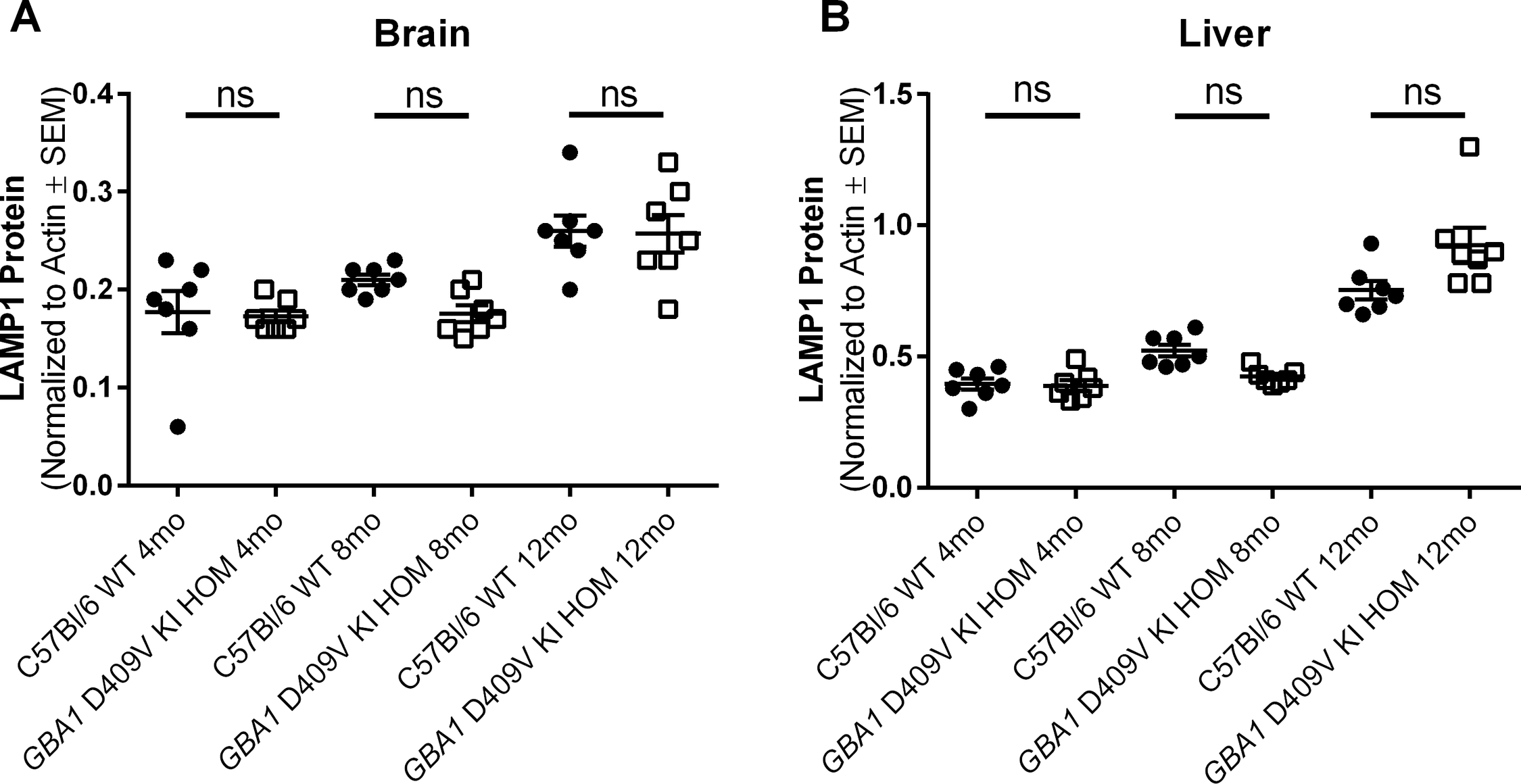

Supplement: S1 Fig — (A-B) Lamp1 levels were measured by Western blot in brain and liver homogenate from C57Bl/6 wild type (WT) mice and GBA1 D409V KI homozygous (HOM) mice at 4, 8, and 12 months of age (n = 7/group). Levels of Lamp1 were not significantly different between WT and GBA1 D409V KI mice at any age in brain (A) or liver (B) tissue. There is an age-related increase in Lamp1 appearing at 12 months of age, with statistically significant differences in the brain and liver of GBA1 D409V KI mice and the liver of WT mice. Lamp1 protein levels are normalized to β-actin as the housekeeping protein. Brain LAMP1 (A) measured as follows: WT 4mo 103.6±3.35, HOM 4mo 98.95±4.40, WT 8mo 124.8±3.11, HOM 8mo 130.7±3.40, WT 12 mo 93.27±2.14, HOM 12mo 121.80±1.98. Liver LAMP1 (B) measured as follows: WT 4mo 0.35±0.02, HOM 4mo 0.37±0.02, WT 8mo 0.30±0.02, HOM 8mo 0.32±0.02, WT 12 mo 0.33±0.01, HOM 12mo 0.48±0.04. Abbreviations: LAMP1, lysosomal associated membrane protein 1; GBA1, gene encoding glucocerebrosidase; WT, wild type; HOM, homozygous; KI, knockin; SEM, standard error of the mean; mo, month; ns, non-significant (p > 0.05). (TIF) [file pone.0252325.s001.tif]

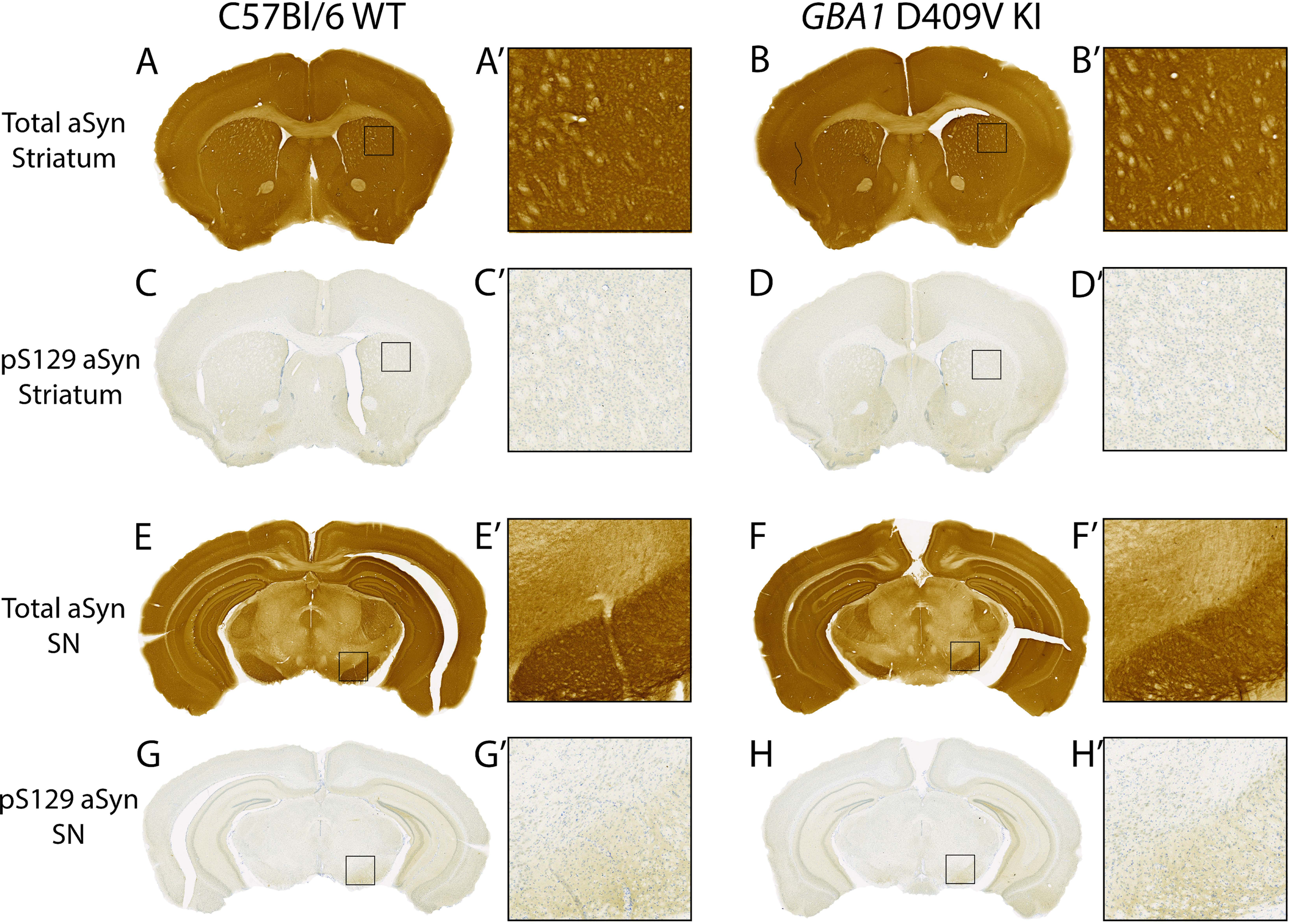

Supplement: S2 Fig — Representative images of immunohistochemical staining for total alpha-synuclein (aSyn; A-B, E-F) and phosphorylated S129 aSyn (pS129 aSyn; C-D, G-H) in C57Bl/6 wild type (WT; A, C, E, G) and GBA1 D409V KI homozygous (HOM; B, D, F, H) mice reveal no overt differences in total aSyn or pS129 aSyn levels between genotypes in either the striatum (A-D) or substantia nigra (SN; E-H). Primed images (A’-H’) are higher magnification images taken using the 10x objective lens at the region of interest denoted with an inset box (A-H). Abbreviations: aSyn, alpha-synuclein; pS129, phosphorylated serine 129; SN, substantia nigra; GBA1, gene encoding human glucocerebrosidase; WT, wild type; HOM, homozygous; KI, knockin. (TIF) [file pone.0252325.s002.tif]

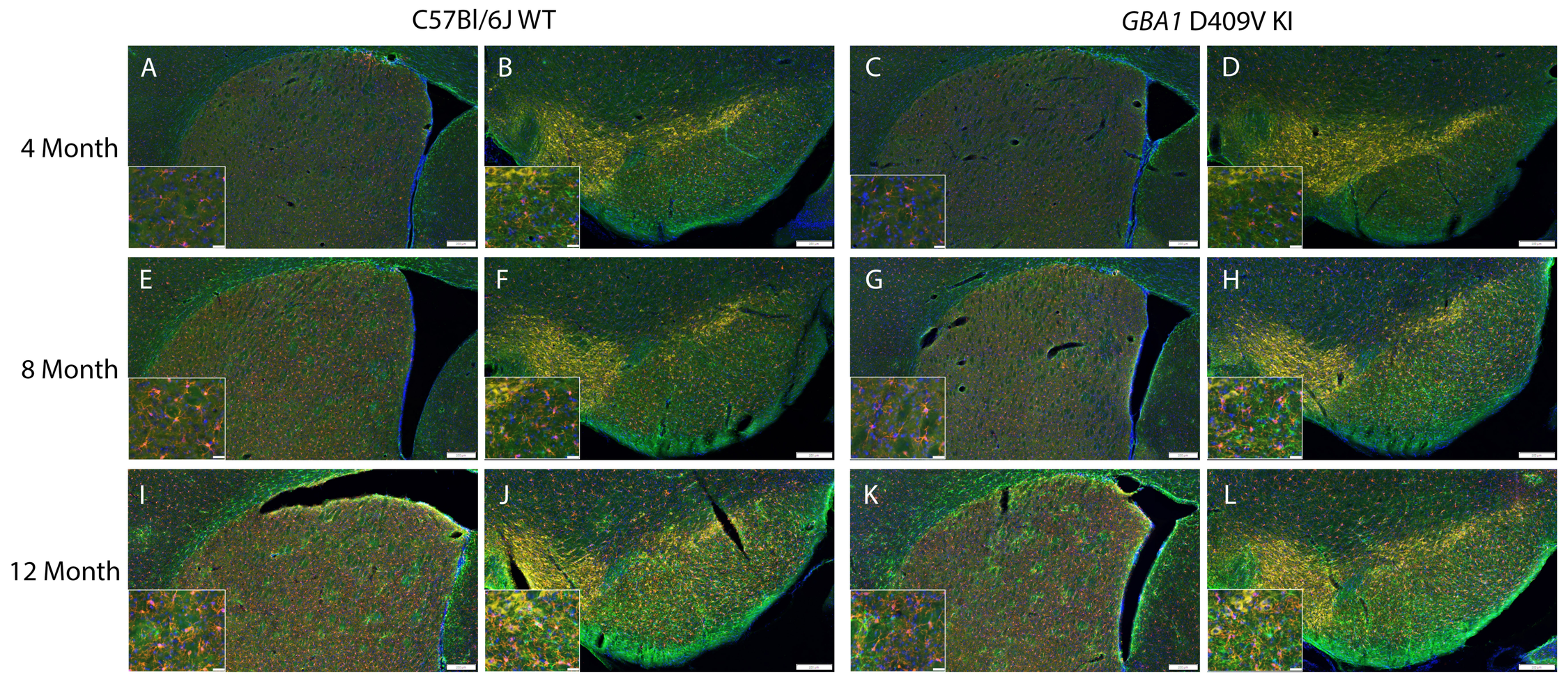

Supplement: S3 Fig — Representative images of immunohistochemical staining for astrocytes using GFAP (green), microglia using Iba-1 (red), dopaminergic neurons using tyrosine hydroxylase (TH; orange), and nuclei using DAPI (blue) in C57Bl/6 wild type (A-B, E-F, I-J) and GBA1 D409V KI homozygous (C-D, G-H, K-L) mice reveal no overt differences in neuroinflammation between genotypes in either the striatum (A, C, E, G, I, K) or substantia nigra (B, D, F, H, J, L). Inset panels are higher magnification images taken using the 40x objective lens. Abbreviations: GBA1, gene encoding human glucocerebrosidase; WT, wild type; KI, knockin. (TIF) [file pone.0252325.s003.tif]
