# Supplementary material for: Decreased glucocerebrosidase activity and substrate accumulation of glycosphingolipids in a novel GBA1 D409V knock-in mouse model
Source: PLoS One. 2021 Jun 9;16(6):e0252325. doi: 10.1371/journal.pone.0252325 (PMC8189458; doi:10.1371/journal.pone.0252325)
Supplement: S1 File — (DOCX) [file pone.0252325.s004.docx]

**S1 File – Raw Data**

*Note: any outliers are denoted in blue italics with an asterisk.*

Figure 2A

| C57Bl/6 WT 4mo | *GBA1* D409V KI HET 4mo | *GBA1* D409V KI HOM 4mo |
| --- | --- | --- |
| 0.803155 | 1.396760 | 1.063203 |
| 0.765823 | 0.762645 | 1.020128 |
| 0.860005 | 0.986290 | 0.881531 |
| 1.110403 | 2.028036 | 0.782637 |
| 1.154885 | 1.029124 | 0.869395 |
| 1.048565 | 1.000520 | 0.858615 |
| 1.066894 | 0.970691 | 0.870802 |
| 1.317756 | 1.306236 |  |

Figure 2B

| C57Bl/6 WT 4mo | *GBA1* D409V KI HET 4mo | *GBA1* D409V KI HOM 4mo |
| --- | --- | --- |
| 0.756562 | 0.936690 | 1.127867 |
| 1.014367 | 0.603100 | 1.098646 |
| 1.142674 | 1.090219 | 1.121308 |
| 1.086397 | 1.216843 | 0.898794 |
| 0.894204 | 1.012345 | 0.970549 |
| 0.993076 | 1.119901 | 1.355179 |
| 0.905452 | 1.659995 | 1.225378 |
| 1.207269 |  |  |

Figure 3A

| C57Bl/6 WT 4mo | *GBA1* D409V KI HOM 4mo | C57Bl/6 WT 8mo | *GBA1* D409V KI HOM 8mo | C57Bl/6 WT 12mo | *GBA1* D409V KI HOM 12mo |
| --- | --- | --- | --- | --- | --- |
| 30.1 | 2.7 | 26.568180 | 2.553690 | 29.313300 | 2.757913 |
| 27.3 | 2.7 | 30.480090 | 3.080881 | 28.731700 | 2.976992 |
| 28.1 | 2.7 | 26.850450 | 4.019727 | 27.141310 | 3.135390 |
| 26.5 | 2.4 | 29.225740 | 3.112779 | 27.506220 | 3.833341 |
| 26.6 | 2.3 | 28.721270 | 3.143344 | 26.068780 | 3.192612 |
| 29.7 | 2.3 | 25.696120 | 2.391628 | 27.584830 | 2.049942 |
| 25.1 | 2.6 | 26.029870 | 2.767473 | 26.113460 | 4.082049 |

Figure 3B

| C57Bl/6 WT 4mo | *GBA1* D409V KI HOM 4mo | C57Bl/6 WT 8mo | *GBA1* D409V KI HOM 8mo | C57Bl/6 WT 12mo | *GBA1* D409V KI HOM 12mo |
| --- | --- | --- | --- | --- | --- |
| 95.007980 | 4.295027 | 142.257400 | 1.928347 | 129.600000 | 1.600000 |
| 76.944370 | 3.389629 | 118.849900 | 0.910289 | 102.600000 | 2.200000 |
| 93.860510 | 3.646738 | 89.175810 | 0.962631 | 79.500000 | 6.200000 |
| 107.193000 | 3.076458 | 99.216950 | 1.080743 | 115.800000 | 3.500000 |
| 74.731210 | 4.649370 | 100.026800 | 2.085771 | 82.900000 | 4.300000 |
| 127.616200 | 4.346902 | 125.361600 | 1.829056 | 85.600000 | 2.700000 |
| 80.460710 | 3.887846 | 111.586800 | 1.724116 | 81.500000 | 4.000000 |

Figure 4A

| C57Bl/6 WT 4mo | *GBA1* D409V KI HOM 4mo | C57Bl/6 WT 8mo | *GBA1* D409V KI HOM 8mo | C57Bl/6 WT 12mo | *GBA1* D409V KI HOM 12mo |
| --- | --- | --- | --- | --- | --- |
| 15.60 | 1.43 | 17.20 | 1.28 | 19.01 | 2.43 |
| 15.14 | 1.23 | 15.87 | 1.33 | 18.41 | 2.27 |
| 15.04 | *2.79** | 15.62 | 1.39 | 19.10 | 2.35 |
| 15.84 | 1.39 | 15.38 | 1.39 | 19.09 | 2.32 |
| 15.40 | 1.37 | 16.18 | 1.40 | 17.30 | 2.37 |
| 15.87 | 1.30 | 15.67 | 1.37 | 18.53 | 2.42 |
| 15.87 | 1.23 | 16.04 | 1.38 | 18.82 | 2.44 |

Figure 4B

| C57Bl/6 WT 4mo | *GBA1* D409V KI HOM 4mo | C57Bl/6 WT 8mo | *GBA1* D409V KI HOM 8mo | C57Bl/6 WT 12mo | *GBA1* D409V KI HOM 12mo |
| --- | --- | --- | --- | --- | --- |
| 47.62 | 2.51 | 66.33 | 2.35 | 72.47 | 2.43 |
| 58.03 | 2.08 | 72.40 | 1.72 | 79.61 | 2.53 |
| 57.59 | 2.17 | 75.04 | 2.20 | 75.54 | 2.53 |
| 68.72 | 2.47 | 73.12 | 2.48 | 80.69 | 1.20 |
| 64.30 | 2.01 | 75.02 | 2.04 | 82.60 | 1.12 |
| 68.96 | 1.96 | 58.96 | 1.50 | 83.58 | 0.71 |
| 51.16 | 1.83 | 64.05 | 1.93 | 79.52 | 1.98 |

Figure 5A

| C57Bl/6 WT 4mo | *GBA1* D409V KI HOM 4mo | C57Bl/6 WT 8mo | *GBA1* D409V KI HOM 8mo | C57Bl/6 WT 12mo | *GBA1* D409V KI HOM 12mo |
| --- | --- | --- | --- | --- | --- |
| 11.16066 | 13.20127 | 19.600000 | 18.900000 | 13.400000 | 15.500000 |
| 7.87521 | 11.02993 | 20.400000 | 23.800000 | 16.900000 | 16.500000 |
| 10.22335 | 11.05103 | 21.700000 | 22.100000 | 13.400000 | 20.900000 |
| 11.11593 | 10.82101 | 18.900000 | 22.800000 | 12.100000 | 20.400000 |
| 10.68096 | 12.09467 | 20.900000 | 18.700000 | 15.500000 | 15.200000 |
| 13.73787 | 17.57048 | 20.500000 | 19.800000 | 17.200000 | 22.500000 |
| 13.02393 | 16.64458 | 20.100000 | 25.100000 | 17.700000 | 19.500000 |
|  |  |  |  |  |  |

Figure 5B

| C57Bl/6 WT 4mo | *GBA1* D409V KI HOM 4mo | C57Bl/6 WT 8mo | *GBA1* D409V KI HOM 8mo | C57Bl/6 WT 12mo | *GBA1* D409V KI HOM 12mo |
| --- | --- | --- | --- | --- | --- |
| 49.034710 | 233.104500 | 87.700000 | 170.000000 | 82.800000 | 310.200000 |
| 102.658700 | 163.615000 | 95.800000 | 198.000000 | 65.200000 | 310.200000 |
| 107.509900 | 189.890500 | 76.600000 | 373.000000 | 87.000000 | 228.400000 |
| 45.525150 | 204.781400 | 67.100000 | 207.000000 | 86.300000 | 152.100000 |
| 73.052320 | 181.907100 | 70.100000 | 199.000000 | 65.200000 | 310.200000 |
| 57.078350 | 180.786800 | 88.500000 | 238.000000 | 56.000000 | 366.600000 |
| 80.030060 | 130.806400 | 105.000000 | 212.000000 | 49.600000 | 110.300000 |

Figure 5C

| C57Bl/6 WT 4mo | *GBA1* D409V KI HOM 4mo | C57Bl/6 WT 8mo | *GBA1* D409V KI HOM 8mo | C57Bl/6 WT 12mo | *GBA1* D409V KI HOM 12mo |
| --- | --- | --- | --- | --- | --- |
| 81.00100 | 1230.00000 | 70.400000 | 614.000000 | 61.500000 | 812.000000 |
| 82.69700 | 984.96000 | 67.800000 | 627.000000 | 49.400000 | 722.000000 |
| 77.39100 | 1110.00000 | 69.200000 | 801.000000 | 69.100000 | 979.000000 |
| 80.78200 | 982.22000 | 68.800000 | 786.000000 | 47.700000 | 629.000000 |
| 74.44700 | 794.12000 | 62.800000 | 773.000000 | 58.400000 | 773.000000 |
| 74.60800 | 1130.00000 | 60.400000 | 688.000000 | 57.900000 | 828.000000 |
| 81.94900 | 994.42000 | 67.100000 | 683.000000 | 56.700000 | 652.000000 |

Figure 5D

| C57Bl/6 WT 4mo | *GBA1* D409V KI HOM 4mo | C57Bl/6 WT 8mo | *GBA1* D409V KI HOM 8mo | C57Bl/6 WT 12mo | *GBA1* D409V KI HOM 12mo |
| --- | --- | --- | --- | --- | --- |
| 19.899000 | 4600.000000 | 25.900000 | 1764.000000 | 14.500000 | 2620.000000 |
| 27.478000 | 3320.000000 | 24.700000 | 1830.000000 | 16.300000 | 2440.000000 |
| 29.613000 | 3890.000000 | 21.900000 | 2383.000000 | 13.700000 | 2600.000000 |
| 14.385000 | 3150.000000 | 25.600000 | 2071.000000 | 13.900000 | 1630.000000 |
| 24.989000 | 3280.000000 | 21.700000 | 2086.000000 | 12.000000 | 2490.000000 |
| 20.014000 | 3160.000000 | 25.000000 | 2603.000000 | 18.400000 | 2850.000000 |
| 27.465000 | 2720.000000 | 26.500000 | 1763.000000 | 14.300000 | 1410.000000 |

Figure 6A

| C57Bl/6 WT 5mo | *GBA1* D409V KI HET 5mo |
| --- | --- |
| 33.0 | 19.0 |
| 32.7 | 18.4 |
| 32.8 | 17.1 |
| 27.4 | 17.4 |
| 33.4 | 16.9 |
| 34.0 | 17.4 |
| 31.3 | 15.6 |
| 23.8 | 18.2 |
| 32.9 | 18.8 |
| 26.9 | 17.7 |

Figure 6B

| C57Bl/6 WT 5mo | *GBA1* D409V KI HET 5mo |
| --- | --- |
| 105.3 | 63.6 |
| 111.0 | 57.2 |
| 154.2 | 55.1 |
| 199.9 | 50.7 |
| 134.1 | 66.7 |
| 150.5 | 59.7 |
| 142.7 | 59.5 |
| 127.8 | 77.9 |
| 124.2 | 70.0 |
| 112.5 | 51.6 |

Figure 6C

| C57Bl/6 WT 5mo | *GBA1* D409V KI HET 5mo |
| --- | --- |
| 13.1 | 10.7 |
| 12.3 | 9.9 |
| 11.9 | 10.4 |
| 12.0 | 10.3 |
| 19.3 | 9.1 |
| 20.3 | 11.4 |
| 21.8 | 9.9 |
| 17.8 | 20.3 |
| 17.1 | 19.2 |
| 18.3 | 21.9 |

Figure 6D

| C57Bl/6 WT 5mo | *GBA1* D409V KI HET 5mo |
| --- | --- |
| 53.7 | 58.4 |
| 59.2 | 63.8 |
| 47.1 | 110.5 |
| 65.3 | 116.0 |
| 62.2 | 79.4 |
| 65.2 | 49.1 |
| 61.5 | 59.7 |
| 74.0 | 55.8 |
| 85.2 | 57.4 |
| 84.6 | 68.7 |

Figure 6E

| C57Bl/6 WT 5mo | *GBA1* D409V KI HET 5mo |
| --- | --- |
| 38.4 | 41.7 |
| 41.1 | 44.5 |
| 40.6 | 46.5 |
| 37.9 | 45.0 |
| 33.4 | 43.2 |
| 37.0 | 39.8 |
| 36.8 | 48.1 |
| 37.0 | 41.7 |
| 38.6 | 45.4 |
| 38.8 | 45.4 |

Figure 6F

| C57Bl/6 WT 5mo | *GBA1* D409V KI HET 5mo |
| --- | --- |
| 11.5 | 14.0 |
| 10.2 | 13.4 |
| 13.2 | 13.9 |
| 14.9 | 18.1 |
| 13.8 | 11.2 |
| 14.5 | 12.1 |
| 11.4 | 12.9 |
| 12.1 | 12.4 |
| 12.0 | 14.4 |
| 12.8 | 14.1 |

Figure 7

| C57Bl/6 WT 4mo | *GBA1* D409V KI HOM 4mo | C57Bl/6 WT 8mo | *GBA1* D409V KI HOM 8mo | C57Bl/6 WT 12mo | *GBA1* D409V KI HOM 12mo |
| --- | --- | --- | --- | --- | --- |
| 8672.1500 | 6293.9580 | 6380.8570 | 5922.8100 | 7691.4240 | 7030.1490 |
| 5761.8770 | 7121.8690 | 6992.8210 | 7489.0480 | 7786.5010 | 7549.1090 |
| 7645.0570 | 7391.4460 | 6026.7040 | 5996.1400 | 7490.0700 | 9225.5480 |
| 6256.4380 | 7671.2950 | 6115.3840 | 8208.6680 | 11141.3100 | 6734.3360 |
| 7898.4750 | 7274.7670 | 10230.8600 | 7182.2540 | 7239.3100 | 7006.2340 |
| 7854.6490 | 7397.8680 | 7432.9420 | 6009.8780 | 7619.8370 | 7392.7380 |
| 6944.8930 | 8024.0270 | 4990.8660 | 8224.8900 | 6974.9480 | 8382.2530 |
| 8051.9440 | 6926.0800 | 7739.5660 | 7237.1700 | 9123.9190 | 7210.2290 |
| 7533.3130 |  | 8010.2730 | 9598.3260 | 8458.0820 |  |

Figure 8A

| C57Bl/6 WT 4mo | *GBA1* D409V KI HOM 4mo | C57Bl/6 WT 8mo | *GBA1* D409V KI HOM 8mo | C57Bl/6 WT 12mo | *GBA1* D409V KI HOM 12mo |
| --- | --- | --- | --- | --- | --- |
| 9.800000 | 8.430000 | 11.200000 | 12.950 | 3.945 | 10.2650 |
| 7.715000 | 11.400000 | 7.635000 | 10.650 | 9.930 | 10.2750 |
| 8.415000 | 12.000000 | 13.220000 | 5.480 | 16.800 | 10.5500 |
| 8.625000 | 8.665000 | 12.500000 | 12.100 | 8.185 | 8.9250 |
| 8.300000 | 9.950000 | 9.800000 | 12.000 | 9.370 | 9.0900 |
| 11.900000 | 9.865000 | 4.700000 | 6.130 | 8.795 | 11.9700 |

Figure 8B

| C57Bl/6 WT 4mo | *GBA1* D409V KI HOM 4mo | C57Bl/6 WT 8mo | *GBA1* D409V KI HOM 8mo | C57Bl/6 WT 12mo | *GBA1* D409V KI HOM 12mo |
| --- | --- | --- | --- | --- | --- |
| 0.480000 | 0.630000 | 0.530000 | 0.350000 | 0.420000 | 0.470000 |
| 0.480000 | 0.460000 | 0.380000 | 0.400000 | 0.280000 | 0.690000 |
| 0.460000 | 0.380000 | 0.260000 | 0.560000 | 0.230000 | 0.500000 |
| 0.490000 | 0.540000 | 0.280000 | 0.390000 | 0.240000 | 0.710000 |
| 0.330000 | 0.650000 | 0.280000 | 0.390000 | 0.240000 | 0.620000 |
| 0.360000 | 0.440000 | 0.540000 | 0.650000 | 0.320000 | 0.330000 |

Supplemental Figure 1A

| C57Bl/6 WT 4mo | *GBA1* D409V KI HOM 4mo | C57Bl/6 WT 8mo | *GBA1* D409V KI HOM 8mo | C57Bl/6 WT 12mo | *GBA1* D409V KI HOM 12mo |
| --- | --- | --- | --- | --- | --- |
| 0.23 | 0.16 | 0.22 | 0.16 | 0.26 | 0.33 |
| 0.20 | 0.16 | 0.20 | 0.16 | 0.25 | 0.28 |
| 0.19 | 0.17 | 0.21 | 0.15 | 0.24 | 0.23 |
| 0.16 | 0.17 | 0.20 | 0.18 | 0.20 | 0.25 |
| 0.18 | 0.16 | 0.19 | 0.17 | 0.27 | 0.30 |
| 0.06 | 0.19 | 0.22 | 0.21 | 0.26 | 0.18 |
| 0.22 | 0.20 | 0.23 | 0.20 | 0.34 | 0.23 |

Supplemental Figure 1B

| C57Bl/6 WT 4mo | *GBA1* D409V KI HOM 4mo | C57Bl/6 WT 8mo | *GBA1* D409V KI HOM 8mo | C57Bl/6 WT 12mo | *GBA1* D409V KI HOM 12mo |
| --- | --- | --- | --- | --- | --- |
| 0.43 | 0.49 | 0.47 | 0.41 | 0.69 | 0.90 |
| 0.46 | 0.40 | 0.57 | 0.39 | 0.73 | 0.95 |
| 0.39 | 0.42 | 0.61 | 0.41 | 0.66 | 0.78 |
| 0.45 | 0.38 | 0.57 | 0.43 | 0.76 | 0.89 |
| 0.30 | 0.33 | 0.48 | 0.48 | 0.93 | 0.78 |
| 0.36 | 0.34 | 0.46 | 0.44 | 0.70 | 0.87 |
| 0.38 | 0.36 | 0.50 | 0.40 | 0.80 | 1.30 |
